# Longitudinal study of stool-associated microbial taxa in sibling pairs with and without autism spectrum disorder

**DOI:** 10.1038/s43705-021-00080-6

**Published:** 2021-12-18

**Authors:** Christine Tataru, Austin Martin, Kaitlyn Dunlap, Marie Peras, Brianna S. Chrisman, Erica Rutherford, Grace E. Deitzler, Alexandra Phillips, Xiaochen Yin, Kayleen Sabino, Roberta L. Hannibal, Wiputra Hartono, Michelle Lin, Edward Raack, Yonggan Wu, Todd Z. DeSantis, Shoko Iwai, Dennis P. Wall, Maude M. David

**Affiliations:** 1 Department of Microbiology, Oregon State University, Corvallis, OR USA; 2 Departments of Pediatrics (Systems Medicine), Biomedical Data Science, and Psychiatry and Behavioral Sciences, Stanford University, Stanford, CA USA; 3 Second Genome Inc., Brisbane, CA USA; 4 Department of Bioengineering, Stanford University, Stanford, CA USA; 5 Department of Biomedical Data Science, Stanford University, Stanford, CA USA; 6 Department of Psychiatry and Behavioral Sciences, Stanford University, Stanford, CA USA; 7 Department of Pharmaceutical Sciences, Oregon State University, Corvallis, OR USA

**Keywords:** Microbiome, Biomarkers

## Abstract

Autism Spectrum Disorder (ASD) is a complex neurodevelopmental disorder influenced by both genetic and environmental factors. Recently, gut dysbiosis has emerged as a powerful contributor to ASD symptoms. In this study, we recruited over 100 age-matched sibling pairs (between 2 and 8 years old) where one had an Autism ASD diagnosis and the other was developing typically (TD) (432 samples total). We collected stool samples over four weeks, tracked over 100 lifestyle and dietary variables, and surveyed behavior measures related to ASD symptoms. We identified 117 amplicon sequencing variants (ASVs) that were significantly different in abundance between sibling pairs across all three timepoints, 11 of which were supported by at least two contrast methods. We additionally identified dietary and lifestyle variables that differ significantly between cohorts, and further linked those variables to the ASVs they statistically relate to. Overall, dietary and lifestyle features were explanatory of ASD phenotype using logistic regression, however, global compositional microbiome features were not. Leveraging our longitudinal behavior questionnaires, we additionally identified 11 ASVs associated with changes in reported anxiety over time within and across all individuals. Lastly, we find that overall microbiome composition (beta-diversity) is associated with specific ASD-related behavioral characteristics.

## Introduction

Autism Spectrum Disorder (ASD) is a complex neurodevelopmental disorder that occurs in 1 out of every 54 children in the United States [1]. ASD is characterized by a set of social and cognitive impairments and can be influenced by a growing number of both genetic and environmental factors [2, 3]. Environmental factors such as maternal prenatal medication use, maternal health factors, and prenatal infection have been associated with ASD development [4]. ASD is also associated with increased prevalence of gastrointestinal (GI) issues [5, 6]. These GI issues can include symptoms such as chronic constipation, diarrhea, abdominal pain, and potential signs of GI inflammation such as vomiting and bloody stools [7]. Growing evidence suggests that ASD is associated with gut dysbiosis. In animal studies, food-based exposure to *Lactobacillus reuteri* or *Bacteroides fragilis* reduced ASD-like social deficits in mice [8, 9]. Evidence of dysbiosis of the gut microbial community has also been observed in humans with ASD, specifically altered levels of *Bifidobacterium*, *Lactobacillus*, and *Clostridium* species [10, 11]. In addition, fecal microbiota transplants in children with ASD demonstrated improvements in GI and ASD symptoms [12, 13]. Reductions of core-ASD symptoms with probiotic usage have also been observed [14].

Studies show that individuals with ASD have increased intestinal permeability and systemic levels of bacterial metabolites that may contribute to ASD pathogenesis by affecting the nervous system [15, 16]. Furthermore, ASD’s comorbid disorders, such as anxiety and Hypothalamus-Pituitary-Adrenal dysfunction, have been associated with gut dysbiosis [17, 18]. Recent studies have also identified microbial markers associated with the ASD phenotype and/or the diet and lifestyle qualities associated with ASD [10, 19–22]. These studies constitute an important initial step towards understanding the complex role the gut plays in neurodevelopmental disorders, but studies to date have often been limited by small sample sizes, limited data on dietary practices, and, most importantly, a lack of longitudinal data points that account for the high variability of gut microbiome structure over time. Notably, most of these studies also do not include a control cohort of participants matched for environmental variables, which have a tremendous impact on the gut microbiome [23, 24]. These are limitations we attempt to address in this paper.

**Fig. 1:**
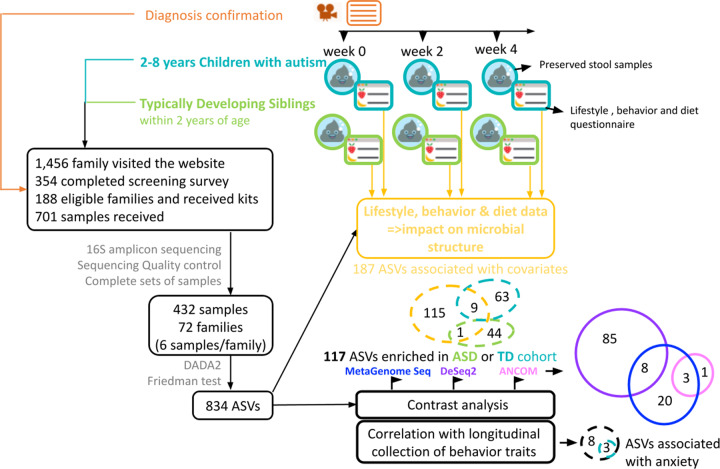
Overall study design. Each sibling pair consisted of one ASD child and their respective TD sibling. Dietary, lifestyle, and other host variables collected can be viewed in Supplementary File 4. The DADA2 pipeline was used to process the 16S V4 amplicon sequences. Samples from sibling pairs with ASD phenotypes unverified by parent reports or home videos were removed, leaving 432 samples. ASVs that significantly varied between timepoints in a Friedman test or were not present in 3% or more of the samples were removed. 117 ASVs were found to be significantly enriched in either the TD or ASD cohort. 11 of those ASVs were identified by more than one of the contrast methods shown above.

The present study aims to characterize the gut microbiome associations in ASD while minimizing issues stemming from limited or single timepoint data. We analyzed 432 stool samples from 72 pairs of siblings diagnosed with ASD and their typically developing (TD) siblings between 2 and 8 years old, and within 2 years of each other, over the course of a month (three timepoints, each separated by two weeks). Each stool sample is accompanied by detailed lifestyle and dietary information (over 100 variables were reported), allowing for a more holistic understanding of ASD and its relationship with the gut microbiome. The present study aims to identify taxa that are differentially abundant between the two cohorts over time, to quantify the predictive value of those microbial features, and to place those microbial associations in the larger context of dietary, lifestyle, and behavioral variables. Figure 1 shows an overview of the study design.

## Results

### Eleven ASVs are significantly associated with the ASD phenotype, as determined by the union of at least two differential analysis methods

Out of 834 total ASVs (Amplicon Sequence Variants, assigned using DADA2), 117 were identified to be significantly different between the ASD and TD cohorts (Supplementary File 1) by at least one of the contrast analysis methods used after normalization and filtration (DESeq2, MetagenomeSeq, and ANCOM, see methods). Out of the 117 ASVs found to be significant across timepoints (Supplementary File 1), 37 belonged to the *Lachnospiraceae* family. *Oscillospiraceae* and *Bacteroidaceae* were the second most represented families with 10 ASVs belonging to each of these families. 93 of the 117 ASVs were detected as significant by DESEQ2, 28 by MetagenomeSeq, and 4 by ANCOM. 45 ASVs were not associated with any lifestyle or dietary variables extracted from the questionnaires. Most notably, 11 ASVs were identified by at least 2 differential analysis methods.

Table 1 summarizes the 11 ASVs with overlapping detection between two contrast methods independently, and their lifestyle/dietary associations if applicable. Two of these were solely associated with the ASD cohort, and no other dietary or metadata co-variate: one from the genus *Holdemania* and one from the family *Lachnospiraceae*. Interestingly, the *Blautia* genus was represented in 3 of the 11 ASVs.

**Table 1:** Eleven ASVs significantly associated with the ASD or typically developing cohorts by two independent contrasts methods.

ASVs taxonomic families, genus, and species are displayed. Some ASVs were unable to be assigned to a single species or genus. ASVs were significant (*q* < 0.05) using the methods displayed. Associated variables significant within a PERMANOVA and correlated significantly to the reported individual ASVs. Arrows indicate positive or negative associations with each variable. (L) refers to variables taken on a longitudinal basis and represent differences in intake near the time of sampling.

^a^Significant permanova variables that significantly differ between cohorts in either a chi-squared test or wilcoxon-ranked sum test. MtgSeq refers to MetagenomeSeq.

**Fig. 2:**
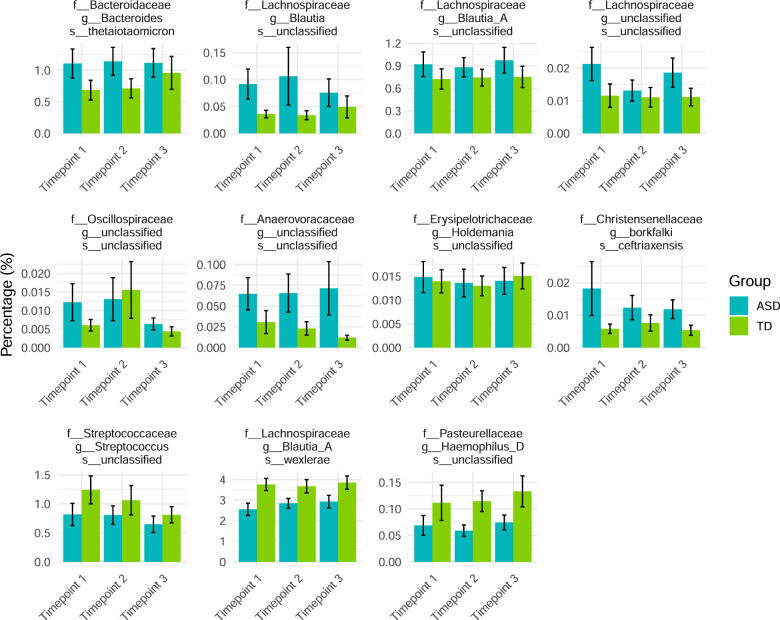
Relative abundance counts of ASVs significantly associated with the ASD cohort in two independent contrast methods. ASVs taxonomic annotation of the 16S amplicon (at the families, genus, and species) and the corresponding relative abundance for the 11 taxa identified in at least two independent contrast methods (ANCOM and/or MetagenomeSeq and/or DESeq2) over the three timepoints.

Figure 2 shows total sum scaled abundance bar plots for the ASVs identified as significant by two methods (CLR normalized counts in Supplementary File 2). The ASV with the highest abundance among the 11 belonged to *Blautia wexlerae* with a relative abundance of almost 4% within the NT and around 2.5–3% within the ASD cohort as shown in Fig. 2. ASVs from *Bacteroides thetaiotaomicron* and a different *Blautia* ASV were among the next highest abundances with values around 0.005–0.01%.

We also performed differential abundance testing using all three contrast methods on ASV counts aggregated by annotated genus (Supplementary File 3). We found many of the differentially abundant ASVs from Table 1 to be members of differentially abundant genera, namely the genera *Bacteroides, Borkfali, Haemophilus, Streptococcus*. Interestingly, the genus *Veillonella* was identified as increased in TD participants by all three analysis methods.

### Demographics, diet, and lifestyle differences between cohorts

We recorded 331 diet and lifestyle variables for each individual participating (Supplementary File 4).

Unsurprisingly, as ASD has been found at a higher prevalence in males [25], 84.7% of the ASD cohort were male as compared to 52.7% of the TD cohort. There were no demographic differences between ASD and TD cohorts as siblings were exclusively documented as the same ethnicity.

A total of 14 of the 331 variables were significantly different between the ASD and TD cohorts. Categorical variables significant in chi-squared tests between cohorts are shown in Table 2 as well as cohort age and C-section birth status. Notably, bowel function and GI symptoms were observed significantly more often in ASD participants, as were special dietary regimes and dietary supplementation (adjusted *p* < 0.05 in Wilcoxon rank-sum tests or two-way repeated anova). Six of these variables were also associated with microbial community dissimilarities using the Bray–Curtis distance metric tested by PERMANOVA. These variables were “dietary restriction”, “dietary supplementation”, “GI symptoms within 3 months”, “GI issues this week”, “habitual fruit consumption”, and “last 2 weeks dairy consumption”. Other variables significantly associated with microbial structure but not significantly different between cohorts can be found in Supplementary File 5 and PCoAs using Bray–Curtis distance and constrained by phenotype and each of the permanova variables with *R*^2^ values above 0.01 are contained in Supplementary File 6. Similar plots using unweighted and weighted UniFrac distance are in Supplementary Files 7 and 8. Extreme consumption rates such as ‘Daily’ or ‘Never’ were, as expected, most easily distinguishable from the microbiome signature.

**Table 2:** ASD and TD comparison of demographic information and significant lifestyle variables.

*Per child*	ASD (*n* = 72)	TD (*n* = 72)	*q* value	Associated with microbial community structure
Probiotic consumption				
Never	15/72	38/72	6.94E−04	No
Rarely	9/72	11/72		
Occasionally	3/72	1/72		
Regularly	6/72	2/72		
Weekly	8/72	6/72		
Several times weekly	11/72	9/72		
Daily	19/72	9/72		
Vitamin B supplementation				
Never	40/72	57/72	1.09E−02	No
Rarely	2/72	7/72		
Occasionally	9/72	1/72		
Regularly	3/72	1/72		
Several times weekly	3/72	1/72		
Daily	15/72	5/72		
Vitamin D consumption				
Never	35/72	47/72	3.25E−02	No
Rarely	1/72	11/72		
Occasionally	3/72	0/72		
Regularly/Weekly	9/72	4/72		
Several times weekly	6/72	4/72		
Daily	1/72	6/72		
Dietary supplement^a^				
True	37/61	14/61	1.40E−03	Yes (*q* = 0.002)
False	24/61	47/61		*R*^2^ = 0.007
Dietary restrictions				
True	27/72	8/72	1.08E−02	Yes (*q* = 0.001)
False	45/72	64/72		*R*^2^ = 0.008
Functional bowel finding^b^				
Tends to have diarrhea	19/72	7/71	7.47E−03	No
Tends to have constipation	13/72	3/71		
Tends to have normal BM	40/72	61/71		
GI symptoms within 3 months				
True	36/72	64/72	2.80E−05	Yes (*q* = 0.004)
False	36/72	8/72		R^2^ = 0.003
Biological sex				
Male	61/72	38/72	1.91E−03	No
Female	11/72	34/72		
Age				
Mean	5.39	4.90	0.15	No
Standard deviation	1.42	2.43		
C-section birth				
True	27/72	27/72	1.00	No
False	45/72	45/72		
Fruit consumption				
Never	25/72	35/72	3.26E−02	Yes (*q* = 0.002)
Rarely	9/72	0/72		*R*^2^ = 0.027
Weekly	11/72	2/72		
Several times weekly	21/72	23/72		
Daily	6/72	12/72		

*Per timepoint*	ASD (*n* = 216)	TD (*n* = 216)		Associated with microbial community structure
Dairy consumption*^M^				
Never/less than once a week	90/203	36/202	3.75E−2	Yes (*q* = 0.001)
3–4 meals per week	33/203	45/202		*R*^2^ = 0.009
7–10 meals per week	42/203	76/202		
Almost every meal	38/203	45/202		
Recent anxiety^M^				
No elevated anxiety	151/203	185/202	1.98E−02	No
Somewhat elevated anxiety	34/203	13/202		
Elevated anxiety	18/203	4/202		
GI issues this week				
True	66/216	9/216	1.02E−11	Yes (*q* = 0.003)
False	150/216	207/216		*R*^2^ = 0.005
Other symptoms this week^**c**^				
True	4/214	23/214	2.76E−03	No
False	210/214	191/214		

^M^Per timepoint dietary consumption and anxiety measures had 13 missing ASD responses and 14 TD responses resulting in an *n* of 203 or 202. *This variable was significant in per timepoint (question answered every 2 weeks) and during initial assessment. The *q* value column is the adjusted *p* value from Wilcoxon-ranked sum tests, chi-squared tests, or two-way repeated-measures ANOVA based on the category of variable. Age and C-section birth status are the only two variables included in this table that were not significantly different between cohorts. Age is listed in years. Racial demographic information and household information not included as they are the same across cohorts for each sibling pair. All participants were from the United States.

^a^Missing 11 TD and ASD responses resulting in an n of 61 out of 72 per cohort.

^b^Missing one TD response.

^c^Missing responses for two timepoints in both ASD and TD.

### Dietary/lifestyle, but not global microbiome compositional features, explain ASD phenotype

To assess the overall associations between lifestyle, microbial factors, and ASD phenotype, we employed logistic regression using different feature sets as follows: (1) Basic (age + sex), (2) Basic + lifestyle/dietary variables, (3) Basic + microbiome features, (4) Basic + lifestyle/diet variables + microbiome features. Microbiome features were calculated as scores along a principal coordinate ordination using Bray–Curtis distance. Additionally, null models were created by replacing features with uniformly randomly distributed noise.

**Fig. 3:**
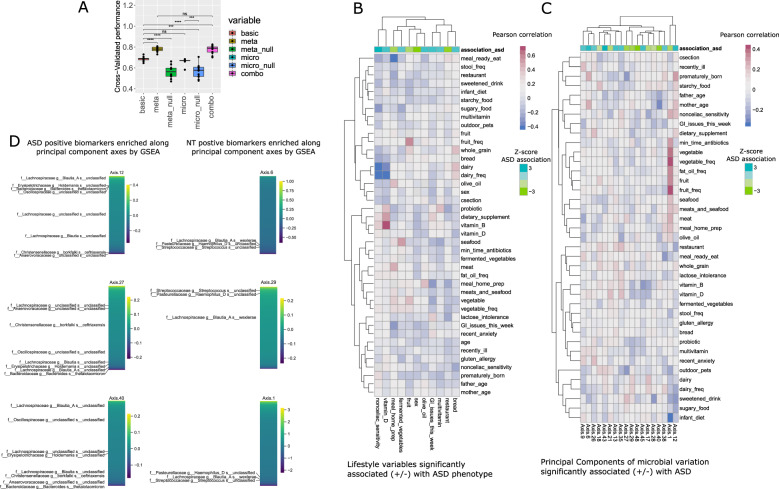
Overall effect size of diet/lifestyle vs. microbial compositional features. **A** Logistic regression models were trained using age + sex (basic), basic + diet/lifestyle features, basic + microbiome features, and basic + diet/lifestyle + microbiome features. Null models included basic + random noise features that matched the range of the original variables. Compared to basic features (AUC = 0.69), diet/lifestyle variables improved cross validated performance significantly (AUC = 0.79) (*p* = 0.004 rank-sum test) while microbiome features did not (AUC = 0.67, *p* = 0.25). **B** Pearson correlation between diet and lifestyle variables significantly related to ASD phenotype within the logistic regression model (vertical) and all other lifestyle variables (horizontal). Columns are annotated by *Z*-score from a slope test within the combo (basic + diet/lifestyle + microbiome) logistic regression model. **C** Pearson correlation between axes of variation (Principal component analysis) that are related to ASD phenotype within the combo logistic regression model (vertical) and all lifestyle variables (horizontal). Columns are annotated by *Z*-score from a slope test within the combo model. **D** ASV abundances are ranked based on their scores across principal components. A set is either the eight biomarkers associated with ASD or the three associated with TD (Table 1). Axes where biomarkers appear significantly skewed to one end or the other (as compared to randomly distributed) as determined by gene set enrichment analysis are represented.

We found that inclusion of lifestyle/dietary variables, but not of microbiome features, significantly improved the explanatory power of a model over basic features (Fig. 3A). Microbiome features did explain phenotype significantly more accurately than random noise variables. A combination of lifestyle and microbiome features did not significantly improve performance over lifestyle features.

Because highly correlated variables are difficult to distinguish using regression models, we calculated the Pearson correlation matrix between all significant lifestyle variables and other lifestyle variables (Fig. 3B). We find that high bread, multivitamin, fermented vegetable, and olive oil consumption, along with GI distress and non-celiac sensitivity, are significant predictors of ASD. Home-prepared meals, as opposed to ready-to-eat meals, were inversely correlated with both ASD phenotype and GI distress.

While the major axes of variation within the gut microbiome did not present additional explanatory power on top of age and sex, some of the axes were statistically significantly related to phenotype. Correlations between axes coordinates and lifestyle variables are found in Fig. 3C. Most notably, a sample’s position along the axes of highest variation (axis1) was associated with the TD phenotype, and scores along this axis correlated with vegetable, fruit, and fat/oil consumption, in addition to meats, seafood, and in general eating home-prepared meals.

Some principal component axes, while not obviously correlated with any lifestyle characteristic, were enriched for the 8 biomarkers associated with ASD or the 3 biomarkers associated with TD (Fig. 2, Table 1). A modified gene set enrichment analysis where a set was considered the eight ASD or three TD biomarkers revealed that scores for biomarkers along particular significant axes were more skewed than would be expected by random chance (gsea *p* < 0.05) (Fig. 3D).

### 11 Taxa correlate with anxiety scores within and across individuals

**Fig. 4:**
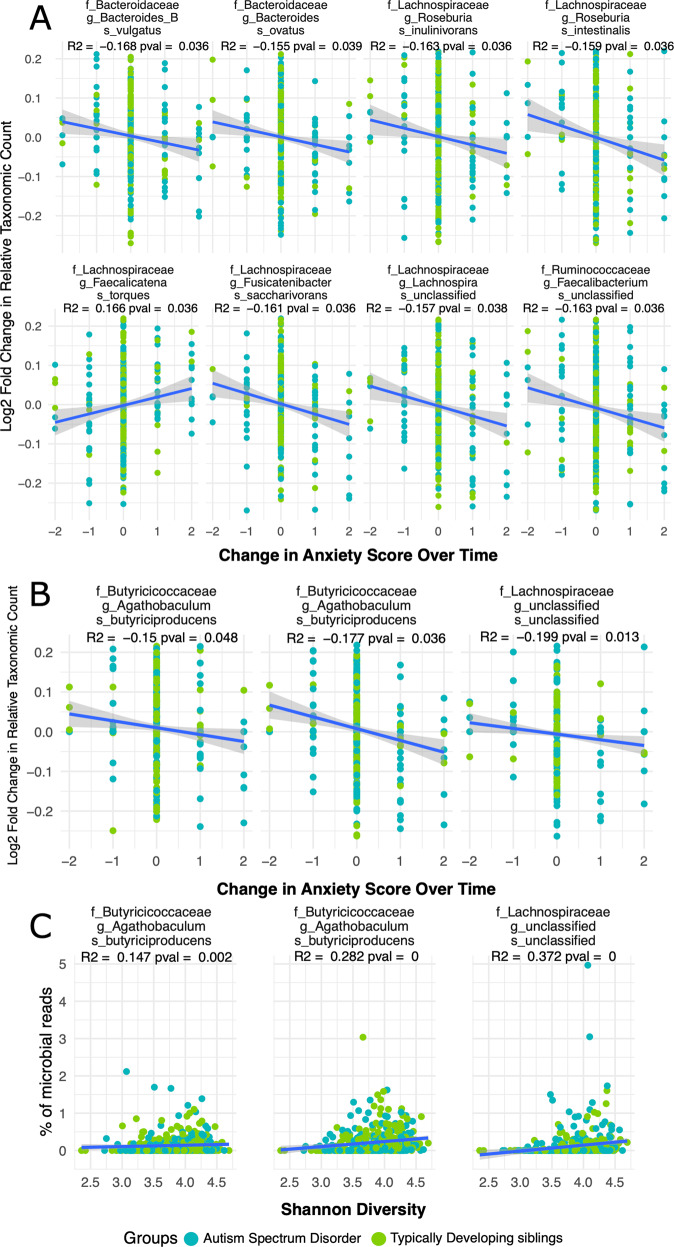
Correlations between changes in anxiety and log2 fold changes in relative taxa abundances. **A** Change of ASVs abundance correlated with changes in anxiety score across the entire cohort. Positive/negative values on the *x* axis signify increases/decreases in anxiety respectively between timepoints within an individual. Positive/negative values on the *y* axis represent an increased/decreased log2 fold change between the relative abundance of an ASV between timepoints within the same individual. *R*^2^ and *p* values represent results from a spearman correlation. **B** ASVs correlated with changes in anxiety scores across both cohorts, and still significant when considering the ASD cohort only. **C** ASVs that correlate negatively with anxiety in the ASD cohort also correlate with alpha diversity (shannon) of samples.

Anxiety in the last 2 weeks before each sample collection was reported by caretakers on a scale of “No elevated anxiety”, “Somewhat elevated”, and “elevated” (0, 1, 2). This metric was used to measure changes in anxiety within the same individual across time, allowing us to fully leverage the longitudinal nature of the data to identify specific ASVs associated with reported anxiety. We found 10 ASVs significantly negatively correlated and 1 ASV positively correlated with increasing anxiety (Fig. 4A, B). Two ASVs from the species *A. butyriciproducens*, well-known for it’s butyrate production, both negatively correlated with anxiety. 6 of the 10 ASVs negatively correlated with anxiety were members of the *Lachnospiraceae* family. Three of the ASVs found correlated with anxiety in the full cohort were similarly correlated with anxiety when considering only the ASD samples (Fig. 4B).

We also found diversity metrics (Chao1, Shannon, FaithPD) to be correlated with ASD severity score (MARA) and age, however, diversity was not significantly different between ASD and TD cohorts (Supplementary File 9).

### Ten behavioral variables are associated with the microbial structure within the ASD cohort

Out of the 14 behavioral questions within the Mobile Autism Risk Assessment (MARA, “Methods” for details) collected in the ASD cohort, 10 were significantly associated with gut microbiome composition (Table 3). Constrained PCOAs using Bray–Curtis distances of DESEQ2 normalized counts were created for each of the significant behavioral variables (Supplementary File 10).

**Table 3:** Significant behavioral variables associated with overall microbial structure in the ASD cohort as determined by a permanova.

Variable	Factor class	Total samples	*R*^2^	*q* value
Childhood behavioral development finding	Numeric	189	0.016	0.005
Plays imaginatively with others	Numeric	213	0.015	0.005
Plays in a group with others	Numeric	216	0.011	0.005
Language ability and use	Numeric	216	0.011	0.005
Sleep pattern finding	Numeric	216	0.01	0.005
Eye contact finding	Numeric	216	0.01	0.005
Repetitive motion	Numeric	216	0.009	0.011
Response to typical sounds	Numeric	216	0.009	0.016
Imitation behavior	Numeric	216	0.008	0.028
Picks up objects to show to others	Numeric	216	0.008	0.033

## Discussion

The present study provides novel insights into the relationship between the gut microbiota and ASD. By leveraging siblings as controls and recording longitudinal gut microbiome, lifestyle, and dietary factors, this work identified differentially abundant taxa between the two cohorts, and identified environmental variables possibly influencing these abundances. We also measure the explanatory power of lifestyle and dietary variables as compared to compositional microbiome features to distinguish between ASD and TD, and identified taxa associated with anxiety changes, a condition comorbid to ASD. Lastly, we find that overall microbiome composition (beta-diversity) is associated with specific ASD-related behavioral characteristics.

### Associating microbial biomarkers with ASD

The set of 117 ASVs identified by any differential analysis method represents the most inclusive analysis and is meant to capture all potentially relevant hits, at the cost of increased expected false positives. In contrast, the 11 ASV set represents the intersection of multiple methods and is expected to contain more robust hits that are reproducible across multiple methods. Among these 11 ASVs, the observed increase in *Bacteroides* annotated*-*ASVs and the decrease of the *Streptococcus* annotated-ASVs in ASD individuals are consistent with findings from previous studies on the genus level [26, 27]. The enrichment of a *Haemophilus* genus member and *Blautia wexlarae* within the TD cohort coincides with findings of increase of *Haemophilus parainfluenzae* and the *Blautia* genus in TD individuals compared to ASD cohorts [27, 28]. In contrast, we also found two other ASVs from the *Blautia* genus were significantly increased in the ASD cohort. Notably, *B. wexlarae* has also been found to be associated with non-celiac gluten sensitivity, a common comorbidity of ASD (we also observed a trend towards gluten sensitivity in our cohort (*q* val 0.01, Supplementary File 11)) [29]. As 16S amplicon studies have limited taxonomic identification, these sub-genus level discrepancies call for deeper phylogenetic characterization, which could be achieved by metagenomic sequencing. We also noted that a member of the *Holdemania* genus was enriched in the ASD cohort, which another study found was associated with ASD children who were classified as “picky eaters” [30]. Association of *Borkfalki ceftriaxensis* and the *Anaerovovracaceae* family with ASD do not seem to have been reported yet in the literature. Among the 117 ASVs detected to be significant in at least one of our methods, *Faecalibacterium, Anaerofustis*, and *Erysipelatoclostridium* ASVs were associated with ASD, and one ASV from *Dialister* with the TD cohort, which is consistent with previous studies, but may represent false-positive hits [27, 31–34].

In addition to ASV level analyses, we also performed differential analysis after aggregating ASV counts into genera. We found many of the differentially abundant ASVs from Table 1 to be members of differentially abundant genera, namely the genera *Bacteroides, Borkfali, Haemophilus, Streptococcus*. Interestingly, the genus *Veillonella* was identified as increased in TD participants by all three analysis methods. This genus is best known for fermenting lactate to propionate and has been observed to be increased in TD controls in other ASD studies as well [19, 22, 35].

### Differentiating between phenotype-based signals and environmental proxies

Dietary selectivity and food sensitivity amongst children with ASD have been widely reported in the literature [19, 36]. Dietary habits and gut microbiome compositions are integrally linked; diet influences microbiome composition and bacteria may influence dietary choices [37]. This study attempted to pinpoint the confounding factors that both impact the microbial structure and are significantly associated with one of the two cohorts within our 4-week-long assessment. Six variables listed in Table 2 were both different between cohorts and associated with gut microbiome composition. As such, these are highly relevant confounding factors that should be considered carefully when untangling the complex relationship between the gut microbiome and ASD.

### Overall effect size of diet/lifestyle vs. microbial signature

While differential analysis allows us to identify specific ASVs of interest, it does not reveal the overall signal strength of the set of variables identified. We used logistic regression models to estimate the explanatory power of environmental variable signatures, and found that lifestyle and dietary features are able to differentiate individuals with ASD from their siblings significantly above a baseline model of sex and age, but that global microbiome compositional features cannot. At the same time, actual microbiome features do provide more explanatory power than random noise, implying that compositional microbiome features may capture some of the influences of diet and lifestyle that are predictive of phenotype. Some axes of gut microbial variation that are not obviously reflective of diet/lifestyle but are still statistically associated with phenotype may be driven by abundance of the reported biomarkers, however, this effect is small compared to the effects of diet.

We emphasize that many of the predictive diet/lifestyle variables are likely informed by a child’s ASD status and therefore cannot be interpreted as directly related to biology. For instance, fermented vegetable consumption and vitamin D supplementation are predictive of the ASD phenotype possibly because parents interested in the intestinal health of their ASD children provide these interventions intentionally.

### The overall microbial structure is associated with self-reported behavioral measures

This study is not the first to report that the microbiota may reflect complex behavioral traits. Multiple animal-animal or human-animal stool microbiome transplant studies have shown that some behavioral traits seem to be mediated by the gut content [38, 39]. In humans, Flannery et al. determined that taxonomic and functional composition of the gut microbiome is associated with behavior and early development in school-aged children [40]. Other studies have also associated microbiome structure with a toddler’s temperament [41]. In ASD specifically, an open-label study showed that microbiota transfer therapy from a neurotypical donor to a recipient with an ASD diagnosis improved GI and behavioral symptoms [12, 13].

Reduction of alpha diversity between children with ASD and a control cohort has been reported in several studies [10, 20, 22, 34, 42] but remains inconsistent across cohorts, especially in sibling studies [43, 44]. Notably in this study, younger participants were more likely to report more severe ASD symptoms and to have a less diverse microbiome, suggesting that discrepancies in age or failure to utilize severity of ASD symptoms in analysis may both be confounding factors and explain some of the discrepancies in alpha diversity metric results across studies.

### Detecting stable microbial signatures over time

We found 33 ASVs that were significant in a single timepoint, but were not consistently different across timepoints. For example, single timepoints implicated ASVs annotated to the species *Faecalibacterium prausnitzii*, *Bacteroides intestinalis*, and *Bifidobacterium bifidum*, all species that have previously been observed as differentially abundant in ASD [31, 34]. Without longitudinal data to account for intra-individual gut microbiome variability, differential analyses are likely to detect false positives. As such, the lack of longitudinal data likely accounts for at least a portion of the inconsistencies observed between gut microbiome association studies [45].

### Anxiety changes over time correlate with changing taxa abundances

Among the ASVs linked to changes in reported anxiety, six ASVs annotated as *Lachnospiraceae* were negatively correlated with anxiety. As mentioned above, the association of the *Lachnospiraceae* family with ASD is widely discussed in the literature; but this family has been reported negatively correlated with ASD comorbid and psychological distress disorders frequently [33, 46–48].

Two ASVs from the same species, *Agathobaculum butyriciproducens*, were negatively correlated with anxiety in the full cohort and within only the ASD cohort. This species is known for heavy butyrate production, which has been demonstrated to decrease gut inflammation and enhance gut epithelial barrier integrity [49, 50]. Given the prevalence of GI issues and leaky gut in ASD [51], the inverse relationship between these butyrate producers and anxiety may be related to some of the GI and core symptoms we observe in ASD. Furthermore, the same three ASVs were positively correlated with the alpha diversity indices in the ASD cohort, suggesting that these taxa are among those lost in lower diversity guts.

While a non-traditional three-point scale was used to measure anxiety, our results are consistent with association scales identified using the more widely used Beck Anxiety Inventory. One study using single timepoint data from 73 individuals reports significant Spearman correlation coefficients of around 0.3 when correlation individual taxa to anxiety metrics across individuals, as compared to our reported coefficients of around 0.16 [52].

### Further considerations and future directions

Despite the number of longitudinal samples and wealth of dietary information, 16S rRNA amplicon analysis can only provide a superficial identification of stool-associated microbial structure. Discrepancies in the species and strain identification could be reduced using shotgun metagenomic sequencing. Paired with multi-omics analysis, such studies would help pinpoint specific mechanisms beyond microbial structure associations.

This paper presents one of the largest longitudinal studies implemented in ASD. But with each sampling separated by two weeks, our sampling only represents a small snapshot of time within an individual’s gut microbiome (one month). Gut microbiomes can fluctuate based on the season due to differences in dietary habits during these times and this effect can be seen to some degree in our study. We attempted to mitigate this issue by sampling sibling control at the same time, but observing a longer collection period would help to maximize the signal/noise ratio.

Previous literature suggests that TD siblings of children with ASD may not constitute a perfect control group, as they tend to exhibit microbiome compositions between their own siblings and unrelated neurotypical controls [22, 53]. Therefore, the ASVs identified in this study are likely to be biased towards precision (confidence that observed difference is related directly to phenotype) rather than sensitivity (confidence that all possible differences have been detected). This study design is likely to have limited the potential biasing effects of enterotypes (distinct community types that stratify individuals) in differential analysis as well [54]. Because sibling pairs are likely to display the same enterotype, a brief investigation into the *Bacteroides* to *Prevotella* as well as *Firmicutes* to *Bacteroidetes* ratios amongst participants suggests an even spread of this variable between phenotypes (Supplementary File 12).

In addition, ANCOM authors have released ANCOMBC, a newer version of the ANCOM than the ANCOM2.1 used in this study [55]. While there may be slight benefit from using ANCOMBC, we do not expect a drastic change to our conclusions by using ANCOMBC as only 4 of the 117 taxa were significant through ANCOM2.1.

Finally, the lifestyle and dietary information collected from participants was self-reported. While we had more objective measures of verifying the ASD phenotype, the validity of the dietary and behavioral measures collected in this study can vary between families, and even within families if the caregiver changed between samplings. Again, we believe that potential reporting inaccuracies were partially mitigated by the fact that the caregiver answered questions for both the ASD child and their TD sibling for each timepoint.

## Conclusion

In the present study, we report 11 microbial taxa consistently associated with ASD in one of the largest longitudinal gut microbiome datasets collected on paired siblings to date. This large sample size was coupled with comprehensive metadata composed of over 100 dietary, lifestyle, and ASD-related variables, and allowed us to identify 6 potential confounding variables within this dataset: habitual fruit and dairy consumption, dietary supplementation or restrictions, and GI symptoms within the past week or last 3 months. In this study, we pinpointed which of the ASVs that significantly differ between the two cohorts are also associated with these factors, providing additional context into what might be driving these associations. We determined that compositional microbiome features do not add extra information above a baseline of age and sex, but diet and lifestyle variables significantly increase explanatory power. We also identified taxa, including *A. butyriciproducens*, that were negatively correlated with anxiety changes across both cohorts. These ASVs are strong candidates for further investigation into the mechanisms behind these findings and the relationship between ASD, the gut-brain axis, and the gut microbiome. Lastly, we find the overall microbiome composition (beta-diversity) did associate with specific ASD-related behaviors, implying that the nature of ASD as a spectrum must be fully considered in future gut microbiome studies.

## Methods

### Recruitment and data collection

We recruited families with two siblings, one previously diagnosed with ASD by a health care provider and one TD, via the website microbiome.stanford.edu. In total, 1432 families visited the website. We recruited children between 23 months to 8 years old, and siblings had to be within 2 years of each other. Dietary, lifestyle, demographic, and host health information were collected via an initial and bi-weekly questionnaires (at each collection time) for each individual (see Supplementary File 4). General dietary habits as well as recent dietary intake during the week prior was collected (labeled as longitudinal within Supplementary File 4).

Each sibling provided three stool samples, spaced two weeks apart. While we received 701 samples total, sibling pairs with individuals that were younger than 23 months, were currently being breast-fed or had ASD children that did not meet ASD criteria (See ASD Diagnosis Verification) were removed, leaving a total of 72 sibling pairs consisting of 432 samples from 144 different participants.

### Autism spectrum disorder diagnosis verification

The MARA, a parent-reported behavioral questionnaire designed to screen children who are at high risk for ASD, was collected electronically from ASD participants [56].

Additionally, parents submitted a short video of their child with and child without ASD via encrypted file share to be rated for ASD symptoms by Stanford Institutional Review Board approved raters on a set of 30 behavioral features [57]. Scores across multiple raters were fed to previously published Machine Learning classifiers to predict ASD risk scores [58, 59]. By combining these risk scores with the parent-report screening tool (MARA), as well as parent-reported physician diagnosis, we confirmed diagnosis using majority rules consensus. We excluded (3) children and their TD siblings for whom the consensus did not agree with original parent diagnosis. Please see Supplementary File 13 for expanded methods.

### Stool collection and storage

Every 2 weeks, caretakers of participants collected a sample using a provided toilet collection kit, and shipped it back at room temperature in preservation buffer (Norgen Biotek, ON, Canada). At the initial timepoint, caretakers also collected a second sample that was immediately frozen at home at −20, then shipped back overnight with two ice packs provided to the participants. Once received, stool samples were stored at −80C until processing. Please see Supplementary File 13 for expanded methods.

### DNA extraction, amplification, and sequencing

Before DNA extraction, stool samples were thawed, pelleted, and supernatant was removed. DNA was extracted from the pelleted stool samples using the MagAttract PowerMicrobiome DNA/RNA Kit (Qiagen) on the KingFisher Flex 96 (ThermoFisher), following manufacturer’s instructions. If DNA did not meet quality standards, an additional DNA clean-up procedure was performed with the Zymo ZR-96 DNA Clean-up kit. All samples were quantified via the Quant-iT PicoGreen dsDNA Assay Kit. The 16S rRNA V4 region was amplified with degenerate primers designed against conserved regions of the 16S rRNA V4 gene region, fused with Illumina adapters and indexing barcodes (slight modifications from [60]). The following primer sequences with adapters, pads, and linkers were used:

Forward primer: AATGATACGGCGACCACCGAGATCTACAC TATGGTAATT GT GTGYCAGCMGCCGCGGTAA

Reverse primer: CAAGCAGAAGACGGCATACGAGAT XXXXXXXXXXXX AGTCAGTCAG CC GGACTACNVGGGTWTCTAAT (Where “XXXXXXXXXXXX” represents the Barcode)

PCR products were cleaned-up using AMPure XP beads (Beckman Coulter) and then quantified via the Quant-iT PicoGreen dsDNA Assay Kit (Invitrogen)

Libraries were pooled, and paired-end sequencing (2 × 250 bps) was performed on an Illumina MiSeq using the MiSeq Reagent Kit v2 (500-cycles) and custom sequencing primers. We obtained an average of 157,103 reads per sample (with a min of 23,321 and max is 996,530).

### Sequence processing, filtering, and taxonomic annotation

Raw sequence reads were processed with DADA2 applying default settings for filtering, learning errors, dereplication, ASV inference, and chimera removal [61]. Truncation quality (truncQ) was set to 2. Ten nucleotides were trimmed from each terminus of each read. An average of 156, 246 reads per sample library remained after processing the raw reads. For strain level ASV assignment, ASVs were mapped to an in-house strain database (StrainSelect, https://www.secondgenome.com/platform/data-analysis-tools/strainselect, version 2019 (SS19)) using USEARCH (usearch_global) in the same manner as a recent study by Shah [62]. Please see Supplementary File 13 for expanded methods.

### Statistical analysis

Statistical analysis was performed using R version 3.6.2 using RStudio Server Pro 1.2.5033-1. The following packages were used: shiny 1.5.0, tibble 3.0.2, data.table 1.13.0, devtools 2.3.1, knitr 1.29, tidyr 1.1.0, reshape2 1.4.4, dplyr 1.0.0, ggplot2 3.3.2, pander 0.6.3, DT 0.14, gridExtra 2.3, adegraphics1.0–15, stats, smart 3.4–8, caret 6.0–86, randomforest 4.6–14, ROCR 1.0–11, exactRankTests 0.8–31, nlme 3.1–148, compositions 2.0–0, ggpubr 0.4.0, vegan 2.5–6, MetagenomeSeq 1.28.2, DESeq2 1.26.0, biomformat 1.14.0, phyloseq 1.30.0 and sourced ANCOM2.1 from https://github.com/FrederickHuangLin/ANCOM.git. Full code used for analysis can be found at https://github.com/MaudeDavidLab/M3_Phyloseq_Analysis/tree/manuscript.

### Normalization and taxa filtration

Before filtration, we attained a minimum read depth of 2.3 × 10^4^ reads and a maximum depth of 9.9 × 10^5^ reads. Taxa not present in at least 3% of the samples were removed. Taxa abundances were normalized using DESeq2 or Cumulative Sum Scaling (CSS) depending on the contrast analysis performed [63, 64]. Due to how DESeq2 normalized minimized intra-group variance within families more so than CSS (Supplementary File 14), DESeq2 was used as the primary normalization in our gut microbial community analysis.

In addition, taxa that significantly vary over time within the same individual were removed to increase the chance of identifying taxa directly related to core phenotype characteristics, rather than changes due to diet or season. A Friedman test was used to model ASV abundance as dependent on timepoint for each individual, and ASVs that were significantly related to timepoint (*p* < 0.1) were removed. 64 ASVs were removed from DESeq normalized data, 78 ASVs were removed from CSS normalized data, and 72 ASVs were removed from unnormalized data.

See Supplementary File 13 for complete methods description.

## Supplementary information

Supplementary File 1

Supplementary Files 2,3, 5-10, 12-14

Supplementary File 4

Supplementary File 11
