# Supplementary material for: Longitudinal study of stool-associated microbial taxa in sibling pairs with and without autism spectrum disorder
Source: ISME Commun. 2021 Dec 18;1:80. doi: 10.1038/s43705-021-00080-6 (PMC9723651; doi:10.1038/s43705-021-00080-6)
Supplement: Supplementary file 2 — Supplementary Files 2,3, 5-10, 12-14 [file 43705_2021_80_MOESM2_ESM.pdf]

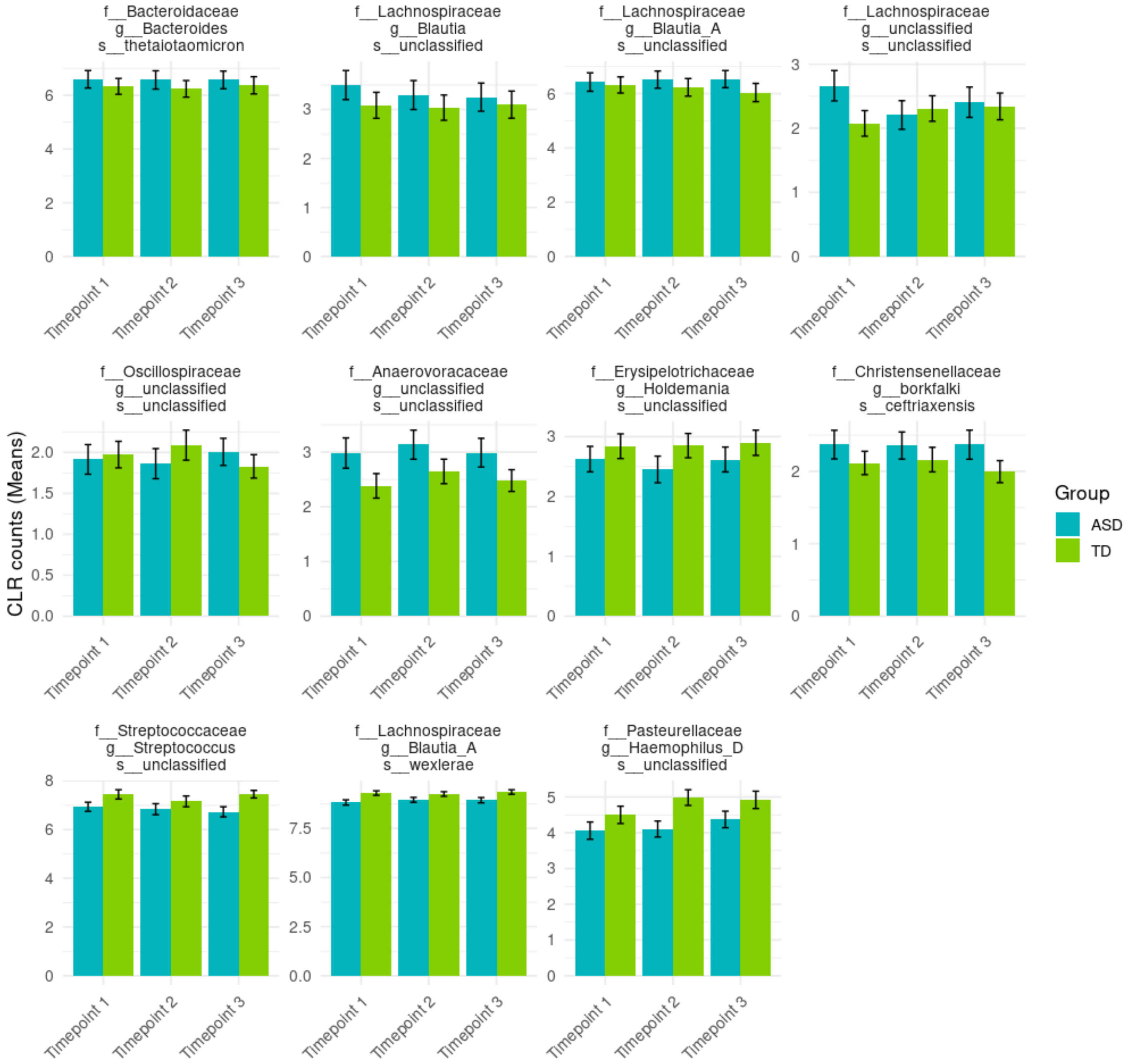

Supplementary File 2: Centered log transform ASVs significantly associated with the ASD cohort in two independent contrast methods. ASVs taxonomic annotation of the 16S amplicon (at the family, genus, and species) and the corresponding centered log transform abundance for the 11 taxa identified in at least two independent contrast methods (ANCOM and/or MetagenomeSeq and/or DESeq2) over the three time points.

A

Order	Family	Genus	Enrichment	Method
Bacteroides	Bacteroidaceae	*Bacteroides*	ASD	DESEQ2
Oscillospirales	*Unclassified*	*Unclassified*	ASD	DESEQ2
Oscillospirales	*Oscillospiraceae*	*Lawsonibacter*	ASD	DESEQ2, Mtgseq
Peptostreptococcales	*Anaerovoracaceae*	*Unclassified*	ASD	DESEQ2, Mtgseq
Coriobacteriales	*Coriobacteriaceae*	*Collinsella*	ASD	Mtgseq
Oscillospirales	*Ruminococcaceae*	*Phocea*	ASD	Mtgseq
Clostridiales	*Unclassified*	*Unclassified*	ASD	DESEQ2
Christensenellales	*Christensenellaceae*	*Borkfalki*	ASD	Mtgseq
Oscillospirales	*Acutalibacteraceae*	*Anaeromassilibacillus*	ASD	Mtgseq
Lactobacillales	*Streptococcaceae*	*Streptococcus*	TD	Mtgseq
Veillonellales	*Veillonellaceae*	*Veillonella*	TD	DESEQ2, Mtgseq, ANCOM
Enterobacterales	*Pasteurellaceae*	*Haemophilus*	TD	Mtgseq, ANCOM

B

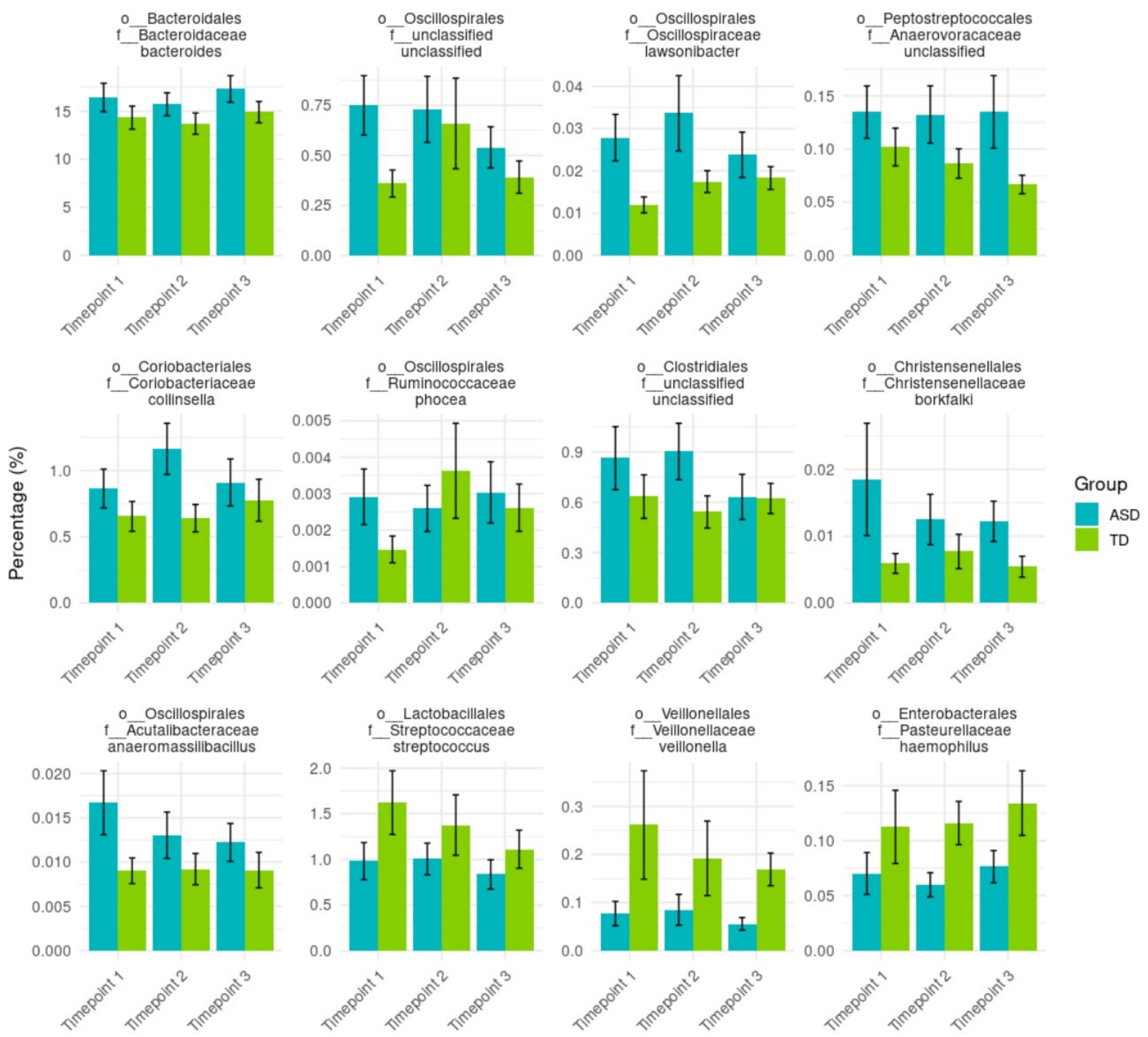

Supplementary Figure 3: Differentially abundant genera in ASD over 3 time points. ASV counts were aggregated by genus level annotation, ASVs that could not be assigned an annotation using the StrainSelect database were removed. Supp. 4A shows the taxonomic annotation of the genus, the phenotype the genus was enriched in, and the analysis method that identified it. Supp. 4B shows the relative percent of that genus in either group ASD or TD. MtgSeq refers to MetagenomeSeq.

Variable	Factor class	Total samples	R^2	q-value
**Vegetable consumption frequency (longitudinal)**	numeric	405	0.027	0.002
**Fruit consumption frequency***	numeric	432	0.012	0.002
**Outdoor pet**	categorical	366	0.011	0.002
**Non celiac gluten sensitivity**	categorical	432	0.011	0.002
**Other GI symptoms**	categorical	366	0.01	0.002
**Sugary food consumption frequency**	numeric	432	0.01	0.002
**Pets in home**	categorical	432	0.009	0.002
**Dairy consumption frequency (longitudinal)***	numeric	405	0.009	0.002
**Toilet trained**	categorical	405	0.008	0.002
**Lactose intolerance**	categorical	432	0.008	0.002
**Dietary restrictions***	categorical	432	0.008	0.002
**Seafood consumption frequency**	numeric	429	0.007	0.002
**Dietary supplement***	categorical	366	0.007	0.003
**Multivitamin**	categorical	432	0.007	0.002
**Fermented vegetable consumption frequency**	numeric	432	0.007	0.002
**GI symptoms within 3 months***	categorical	432	0.006	0.004
**GI issues this week***	categorical	432	0.005	0.003
**GI issues two weeks ago**	categorical	432	0.004	0.013

Supplementary File 5: Lifestyle and dietary features significantly associated with overall microbiome composition (as determined by a PERMANOVA test) but NOT significantly different between the ASD and TD cohort.

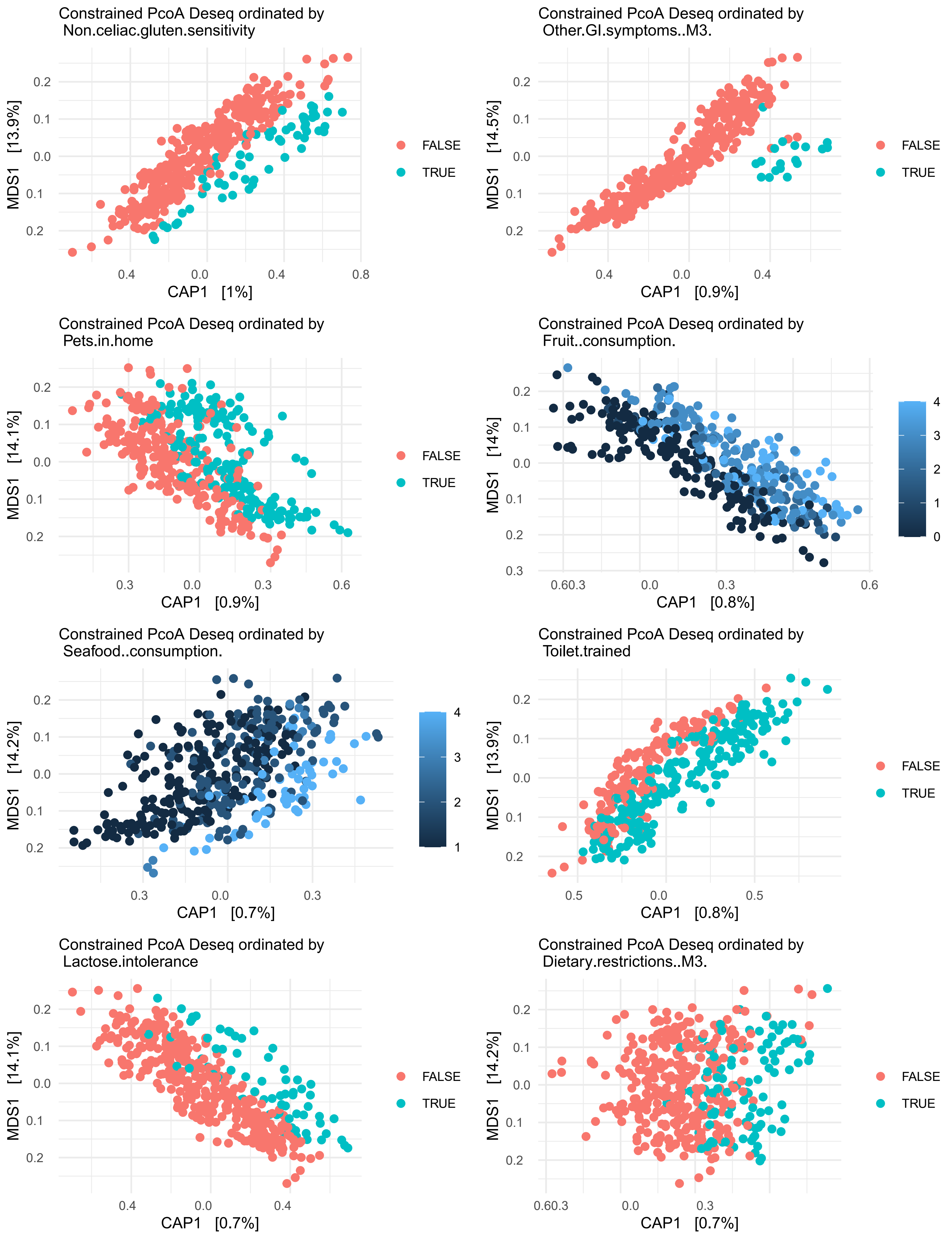

Supplementary File6 : PCoAs using bray curtis distance constrained by phenotype (ASD or TD) along with the variable in each plot title. Colors correspond to the variable in each plot title. Only those variables that were determined to be significantly associated with microbiome composition (PERMANOVA) are depicted.

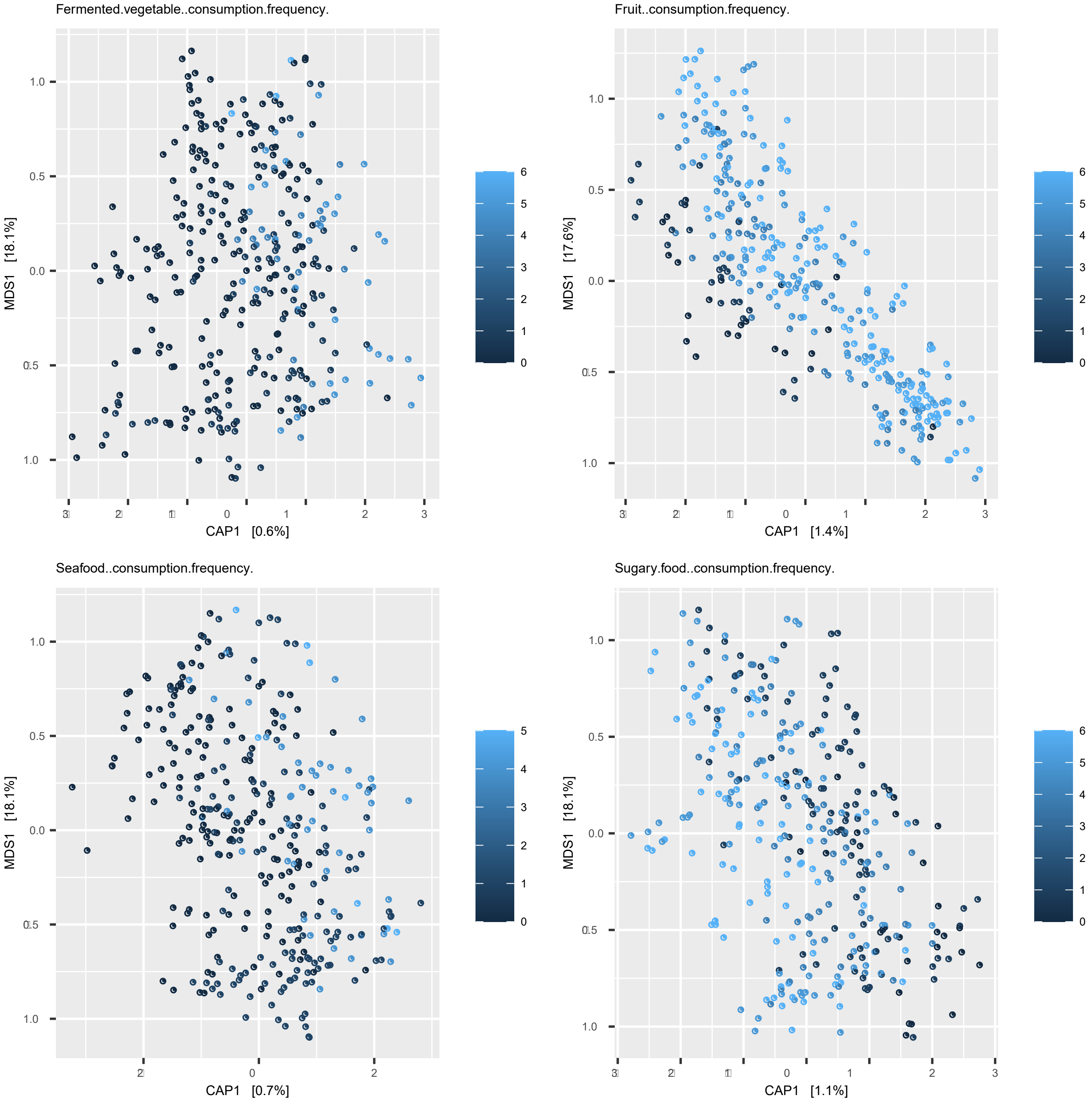
Fermented.vegetable..consumption.frequency.
MDS1 [18.1%]
CAP1 [0.6%]
6
5
4
3
2
1
0
Fruit..consumption.frequency.
MDS1 [17.6%]
CAP1 [1.4%]
6
5
4
3
2
1
0
Seafood..consumption.frequency.
MDS1 [18.1%]
CAP1 [0.7%]
5
4
3
2
1
0
Sugary.food..consumption.frequency.
MDS1 [18.1%]
CAP1 [1.1%]
6
5
4
3
2
1
0

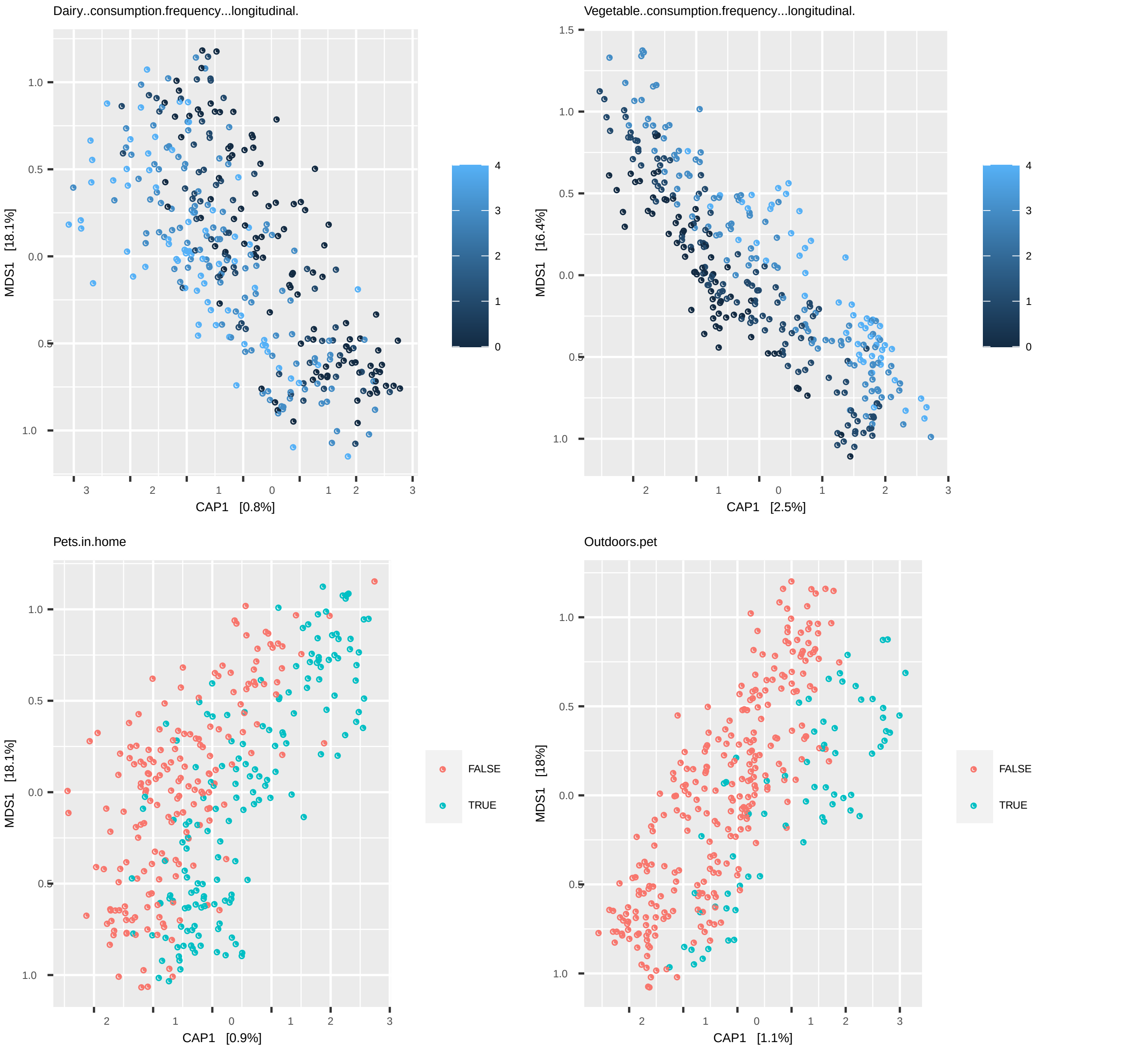
Dairy..consumption.frequency...longitudinal.
MDS1 [18.1%]
CAP1 [0.8%]
0
1
2
3
4
Vegetable..consumption.frequency...longitudinal.
MDS1 [16.4%]
CAP1 [2.5%]
Pets.in.home
MDS1 [18.1%]
CAP1 [0.9%]
FALSE
TRUE
Outdoors.pet
MDS1 [18%]
CAP1 [1.1%]
FALSE
TRUE

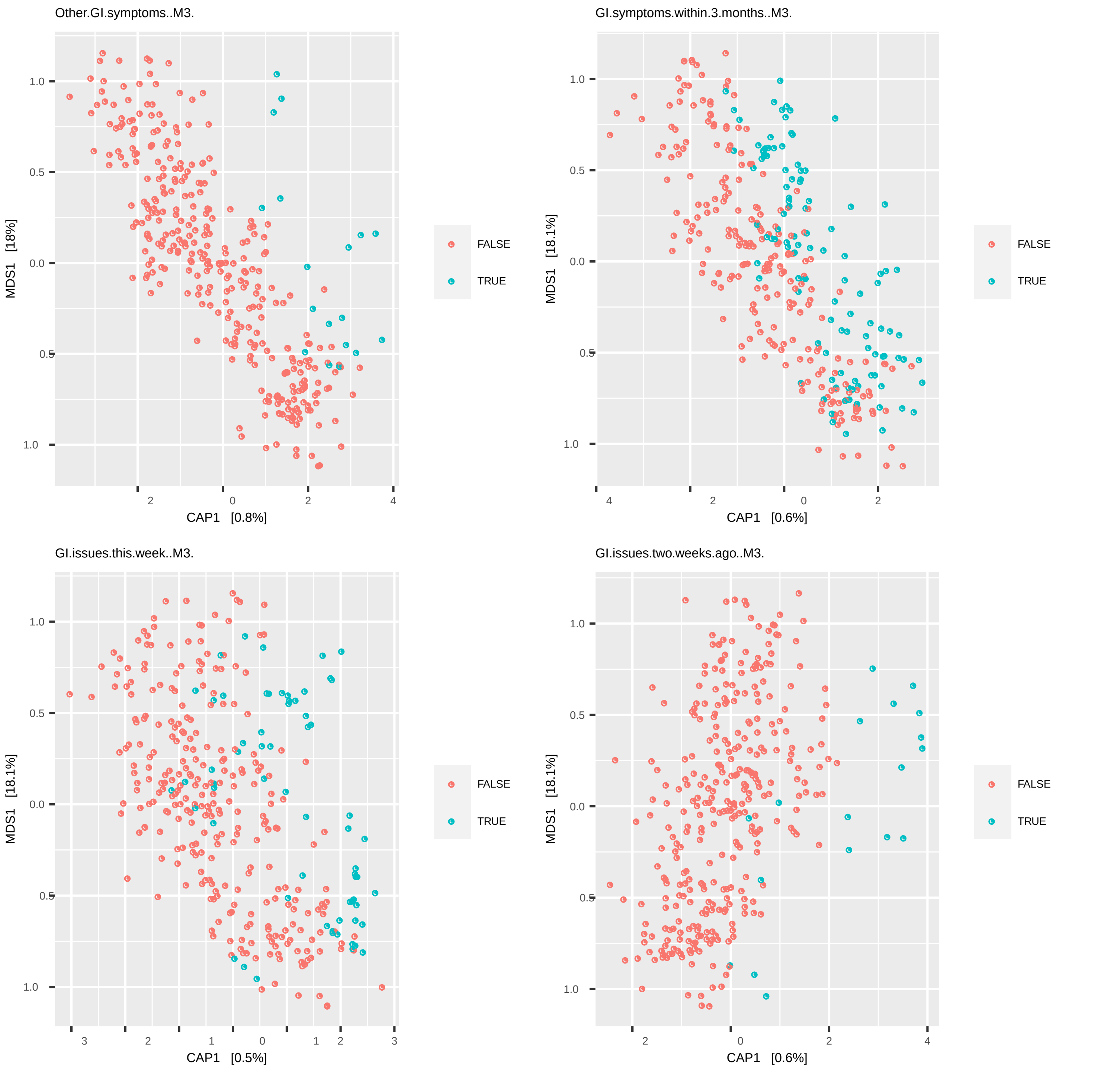
Other.GI.symptoms..M3.
MDS1 [18%]
CAP1 [0.8%]
1.0
0.5
0.0
0.5
1.0
2
0
2
4
FALSE
TRUE
GI.symptoms.within.3.months..M3.
MDS1 [18.1%]
CAP1 [0.6%]
4
2
0
2
FALSE
TRUE
GI.issues.this.week..M3.
MDS1 [18.1%]
CAP1 [0.5%]
3
2
1
0
1
2
3
FALSE
TRUE
GI.issues.two.weeks.ago..M3.
MDS1 [18.1%]
CAP1 [0.6%]
2
0
2
4
FALSE
TRUE

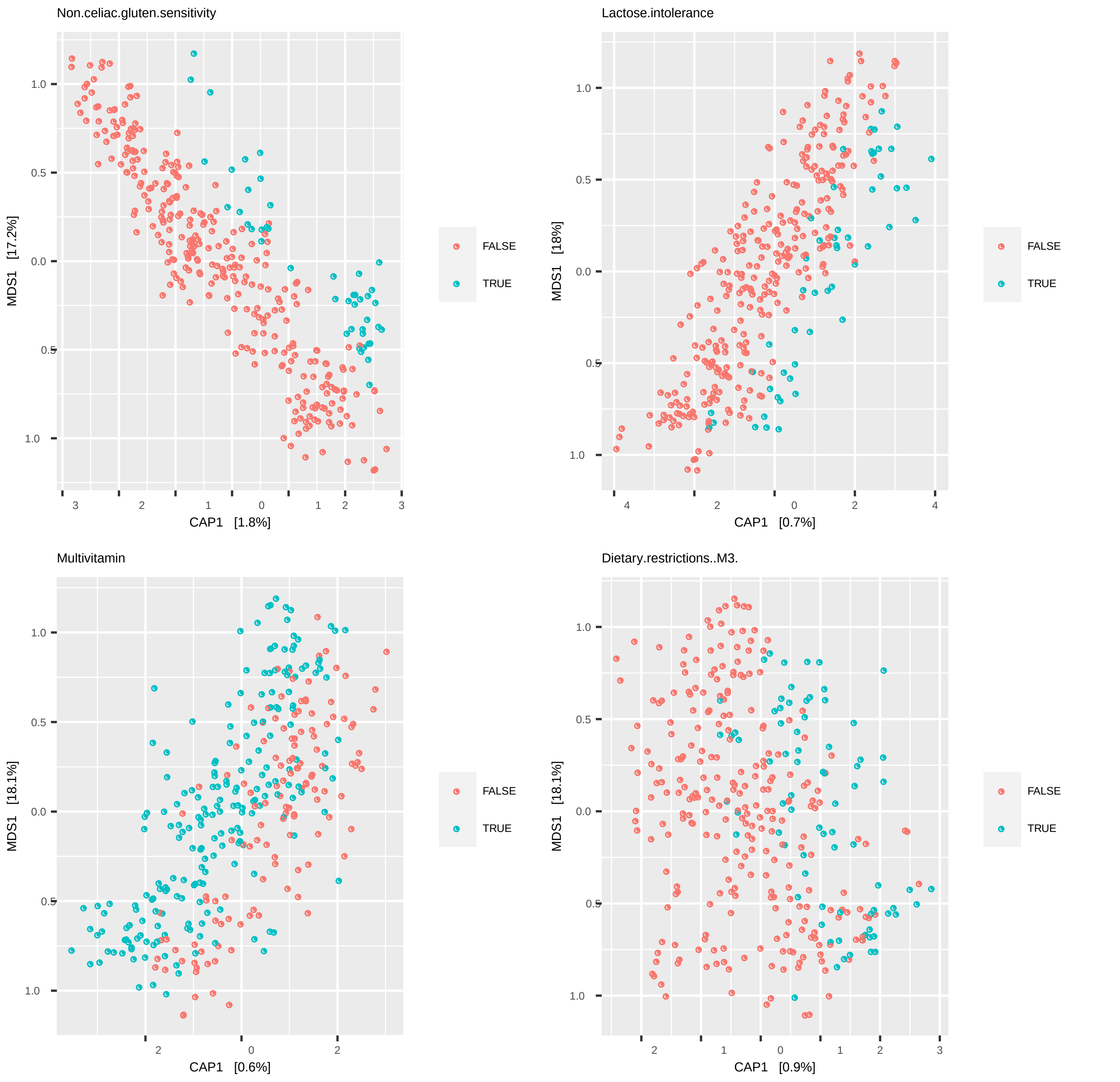

Non.celiac.gluten.sensitivity
MDS1 [17.2%]
CAP1 [1.8%]
1.0
0.5
0.0
0.5
1.0
3
2
1
0
1
2
3
Lactose.intolerance
MDS1 [18%]
CAP1 [0.7%]
1.0
0.5
0.0
0.5
1.0
4
2
0
2
4
Multivitamin
MDS1 [18.1%]
CAP1 [0.6%]
1.0
0.5
0.0
0.5
1.0
2
0
2
Dietary.restrictions..M3.
MDS1 [18.1%]
CAP1 [0.9%]
1.0
0.5
0.0
0.5
1.0
2
1
0
1
2
3
FALSE
TRUE

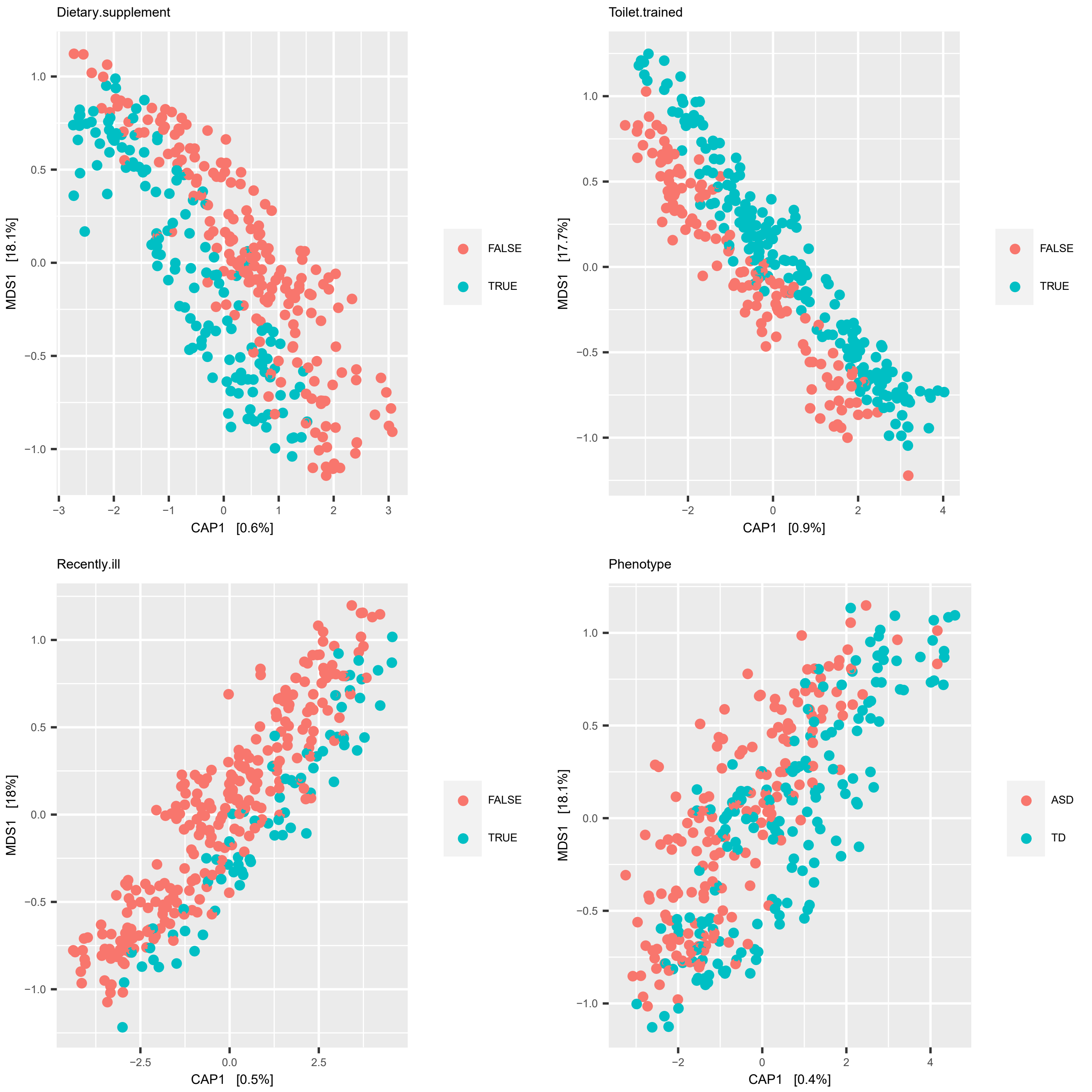

Supplementary File 7: PCoA using weighted UniFrac distances constrained by the variable in each plot title. Colors correspond to the variable in each plot title.

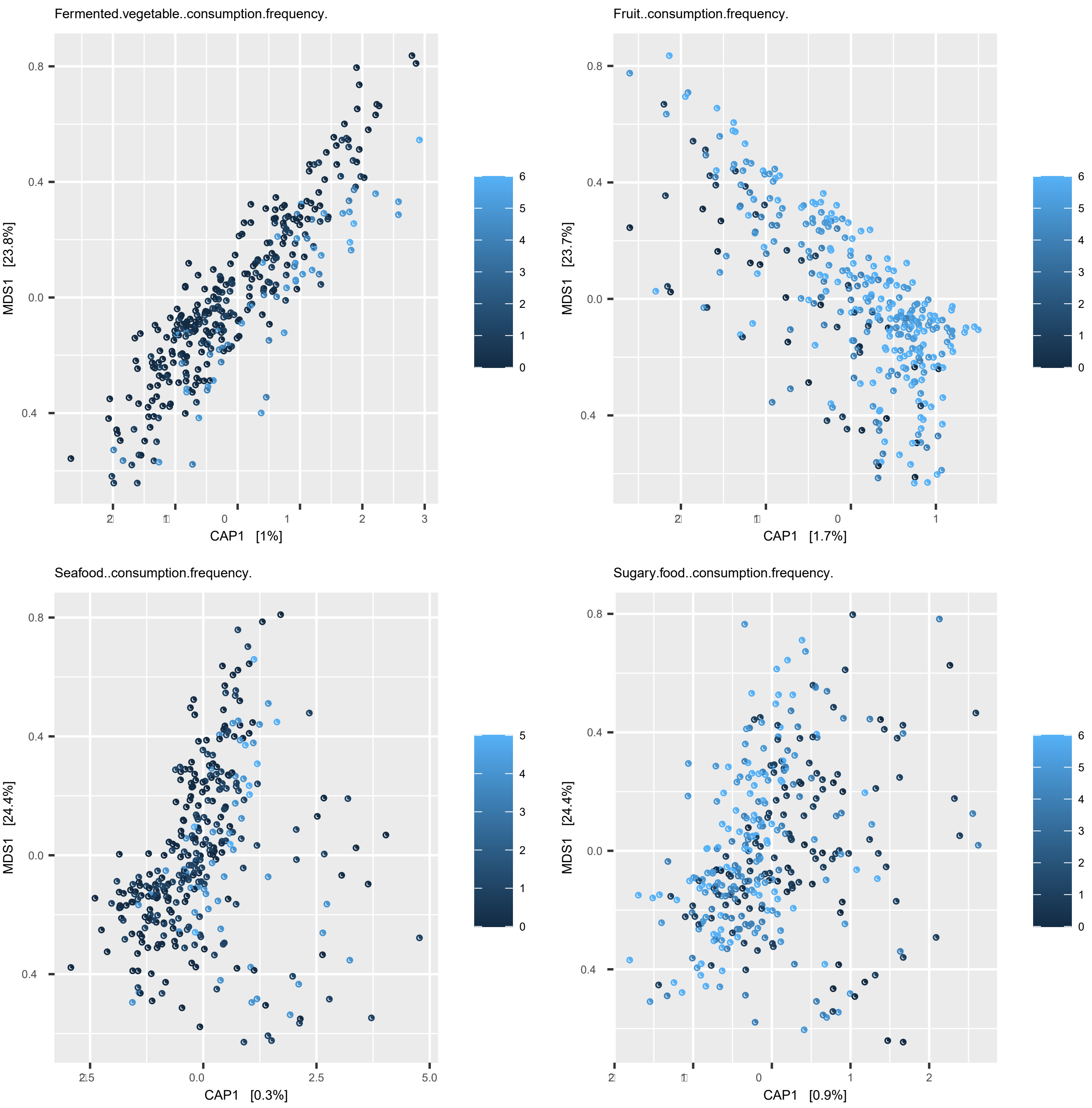
Fermented.vegetable..consumption.frequency.
MDS1 [23.8%]
CAP1 [1%]
0.8
0.4
0.0
0.4
0
1
2
3
6
5
4
3
2
1
0
Fruit..consumption.frequency.
MDS1 [23.7%]
CAP1 [1.7%]
0.8
0.4
0.0
0.4
0
1
Seafood..consumption.frequency.
MDS1 [24.4%]
CAP1 [0.3%]
0.0
2.5
5.0
5
4
3
2
1
0
Sugary.food..consumption.frequency.
MDS1 [24.4%]
CAP1 [0.9%]
0
1
2
6
5
4
3
2
1
0

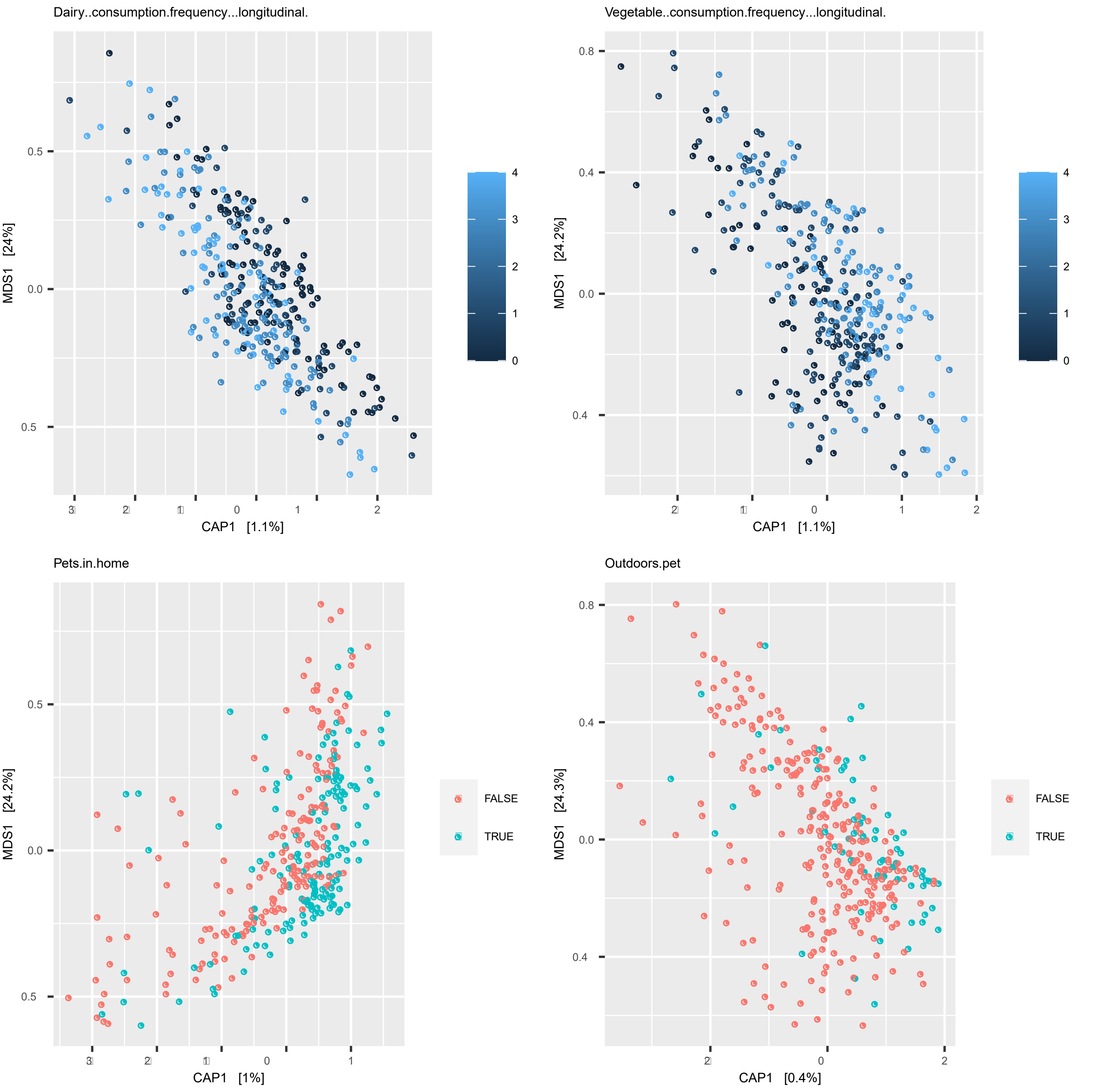

Dairy..consumption.frequency...longitudinal.
MDS1 [24%]
CAP1 [1.1%]
Vegetable..consumption.frequency...longitudinal.
MDS1 [24.2%]
CAP1 [1.1%]
Pets.in.home
MDS1 [24.2%]
CAP1 [1%]
FALSE
TRUE
Outdoors.pet
MDS1 [24.3%]
CAP1 [0.4%]
FALSE
TRUE

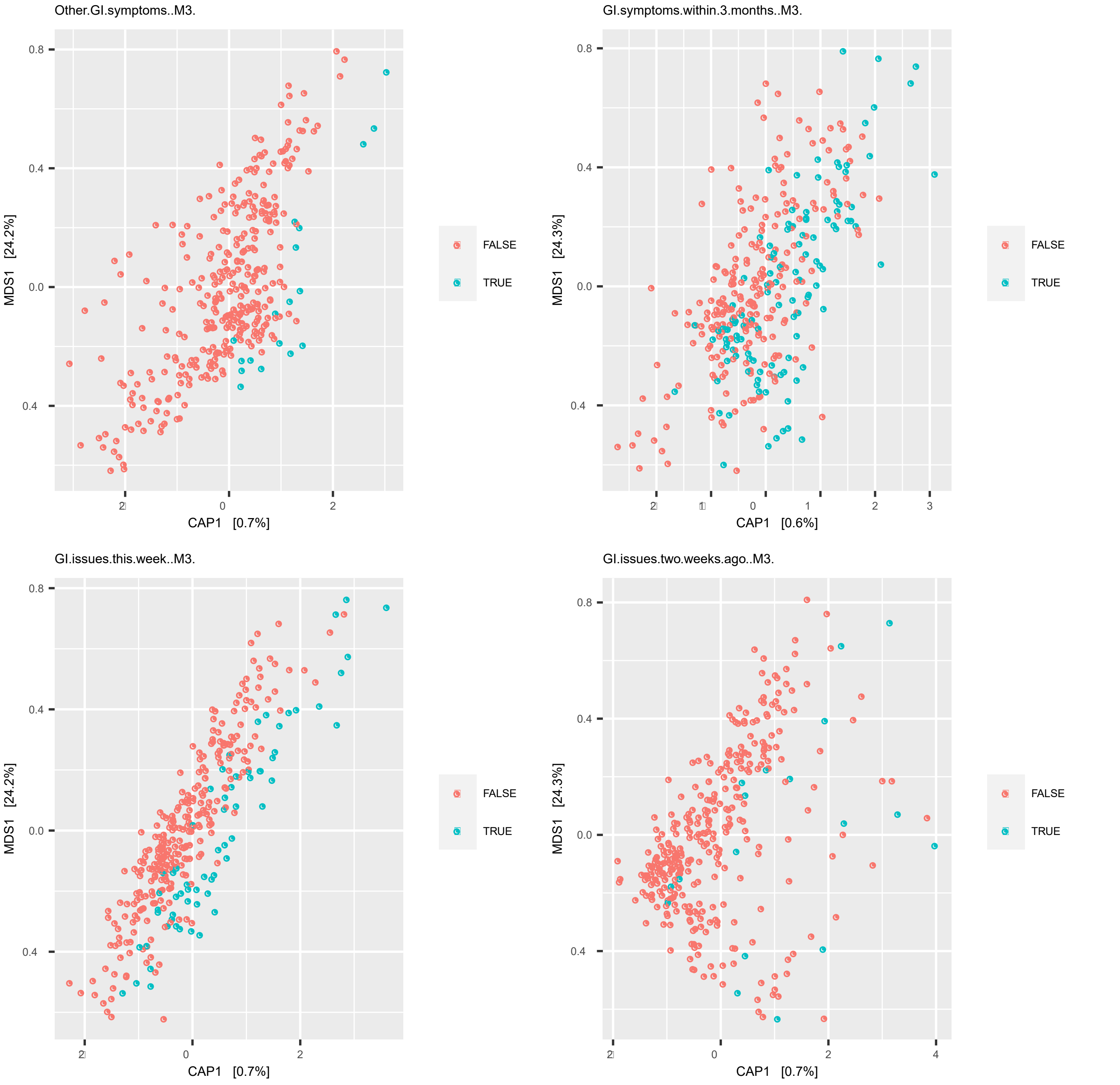
Other.GI.symptoms..M3.
MDS1 [24.2%]
CAP1 [0.7%]
FALSE
TRUE
GI.symptoms.within.3.months..M3.
MDS1 [24.3%]
CAP1 [0.6%]
FALSE
TRUE
GI.issues.this.week..M3.
MDS1 [24.2%]
CAP1 [0.7%]
FALSE
TRUE
GI.issues.two.weeks.ago..M3.
MDS1 [24.3%]
CAP1 [0.7%]
FALSE
TRUE

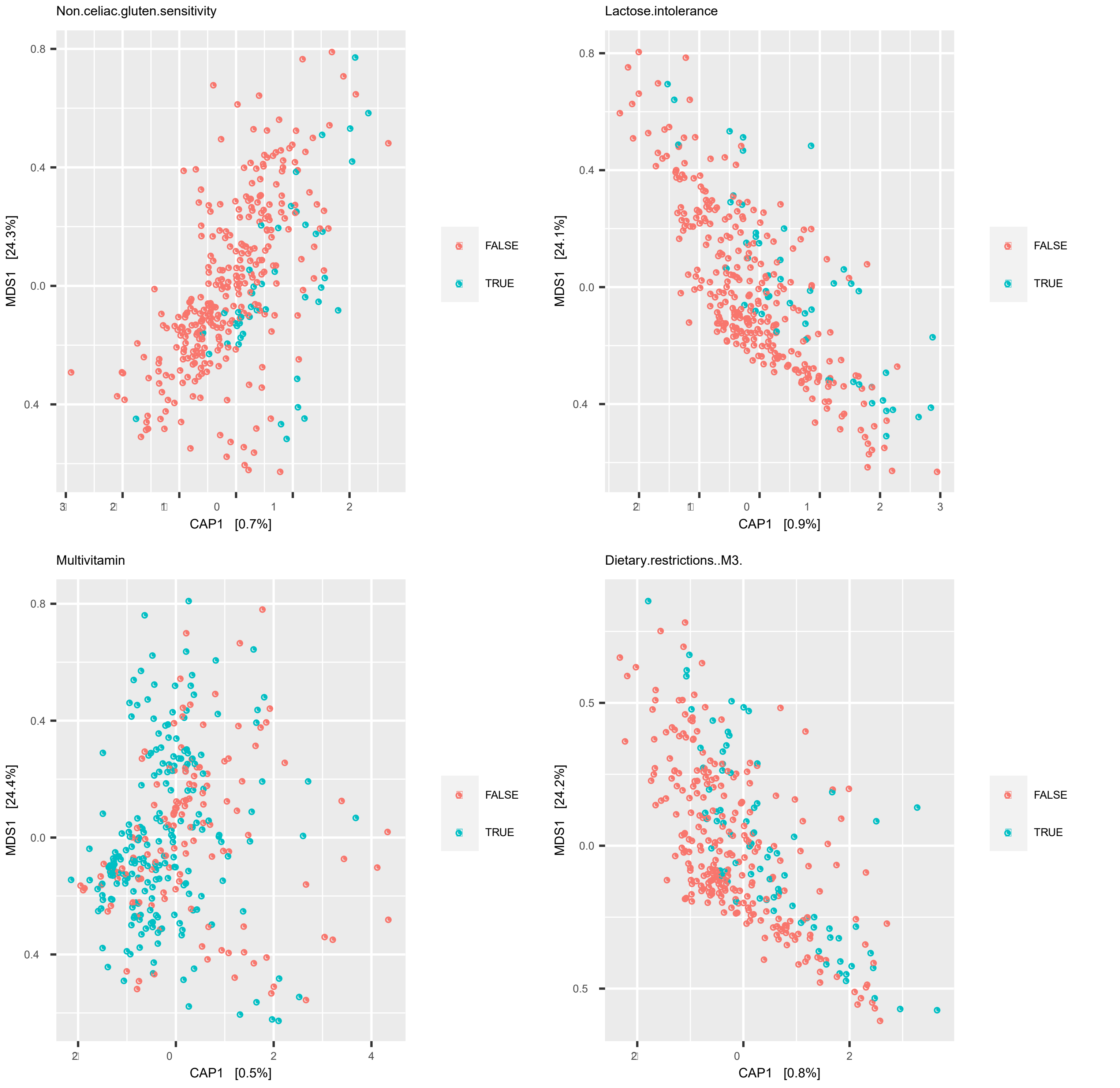

Non.celiac.gluten.sensitivity
MDS1 [24.3%]
CAP1 [0.7%]
Lactose.intolerance
MDS1 [24.1%]
CAP1 [0.9%]
Multivitamin
MDS1 [24.4%]
CAP1 [0.5%]
Dietary.restrictions..M3.
MDS1 [24.2%]
CAP1 [0.8%]
FALSE
TRUE

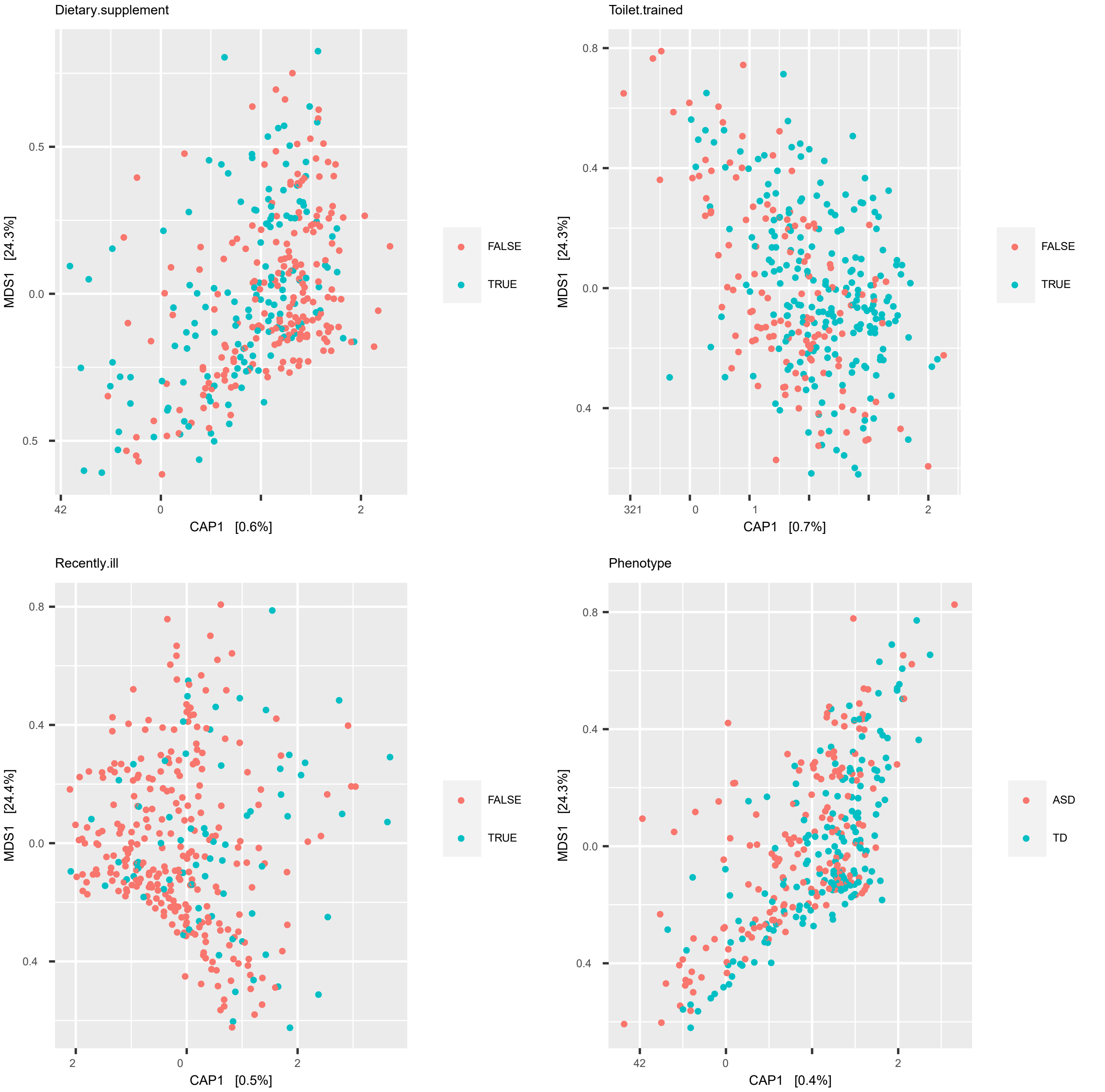

Supplementary File 8: PCoA using unweighted UniFrac distances constrained by the variable in each plot title. Colors correspond to the variable in each plot title

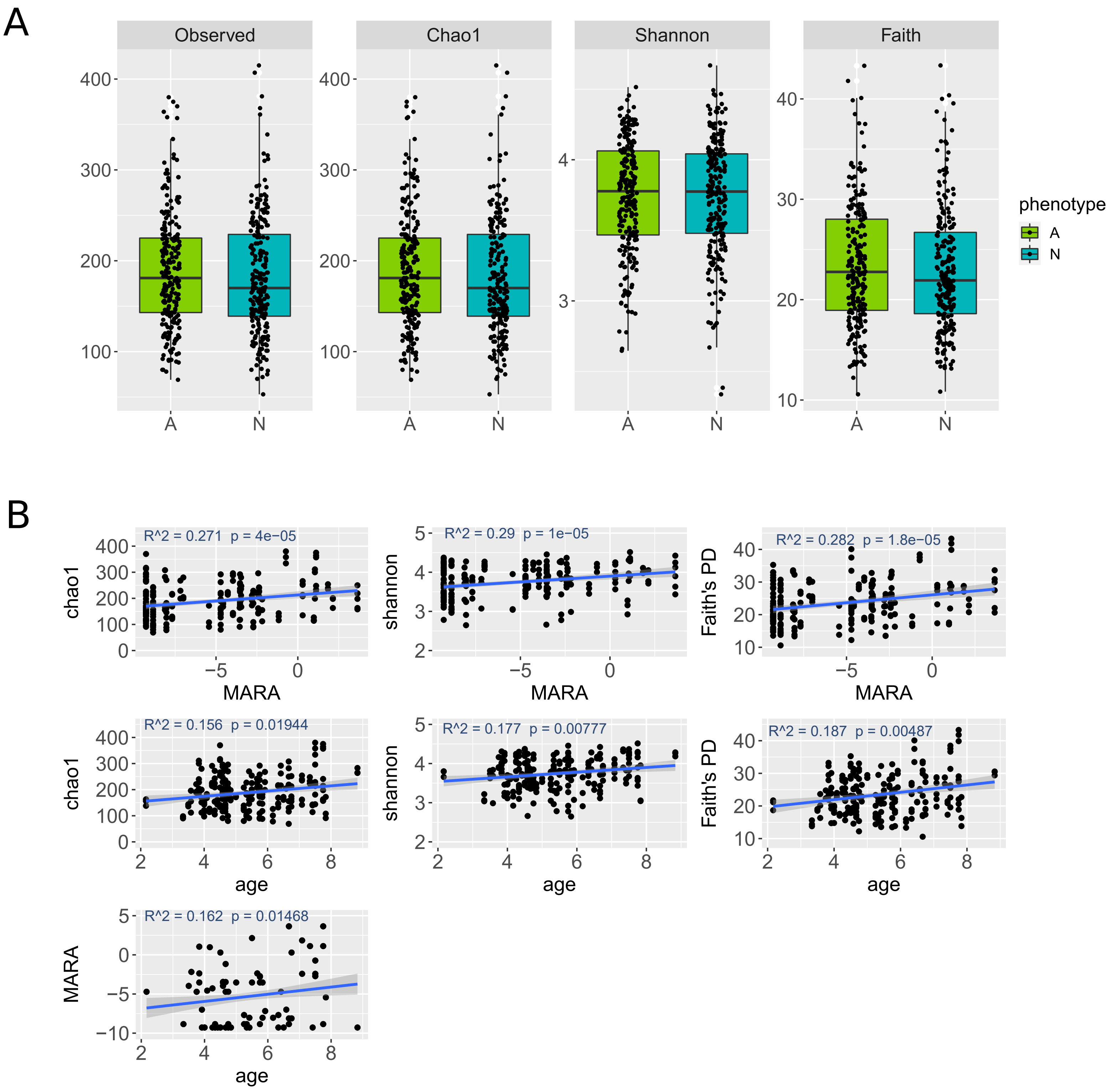

Supplementary File 9:
A) Diversity metrics Chao1, Shannon, and Faith's Phylogenetic Diversity indices, as compared between ASD and TD. None of the metrics showed statistical significance between groups as determined by a t-test and Mann-Whitney rank sum test.

B) Spearman correlations show statistical significance between diversity metrics, MARA score (ASD severity), and age (measured in years). Negative MARA score represents more severe ASD behavioral symptoms.

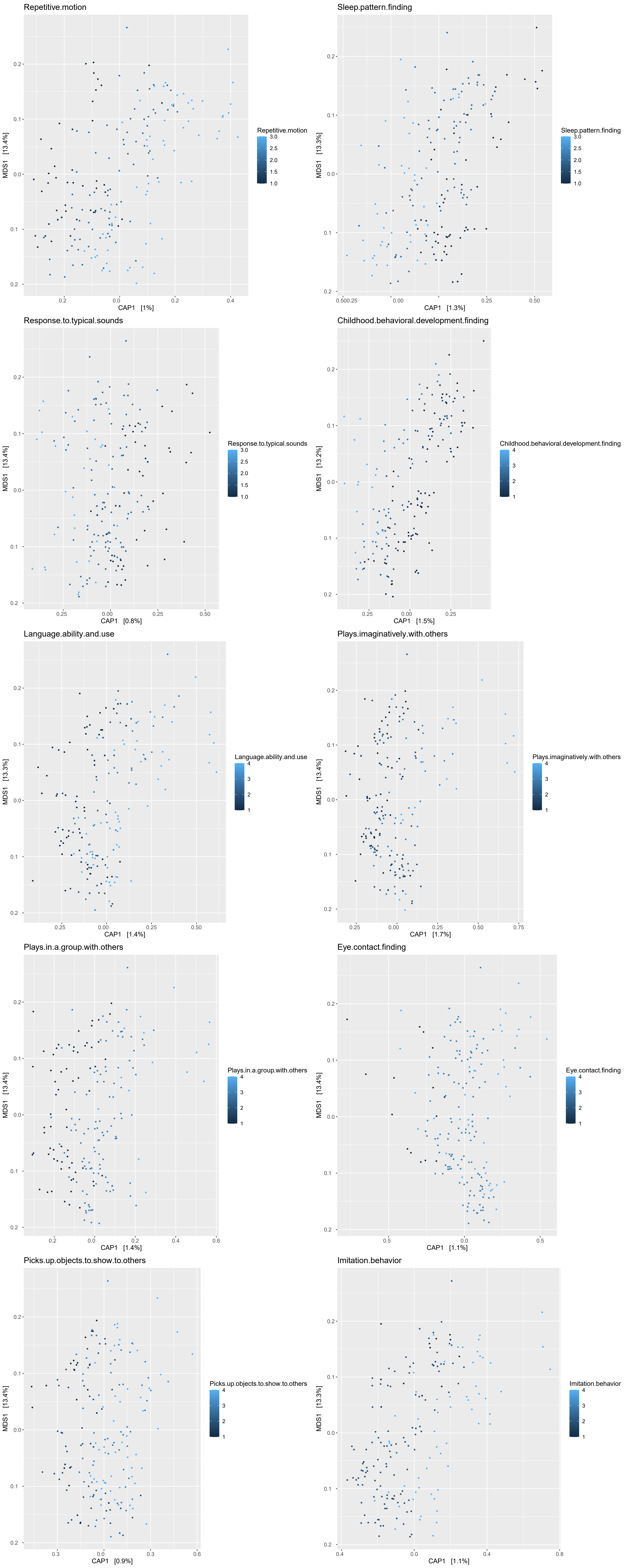

Supplementary File 10: PCoA using Bray Curtis distance and constrained and colored by behavioral traits in the title of each plot. Samples from ASD participants only are used.

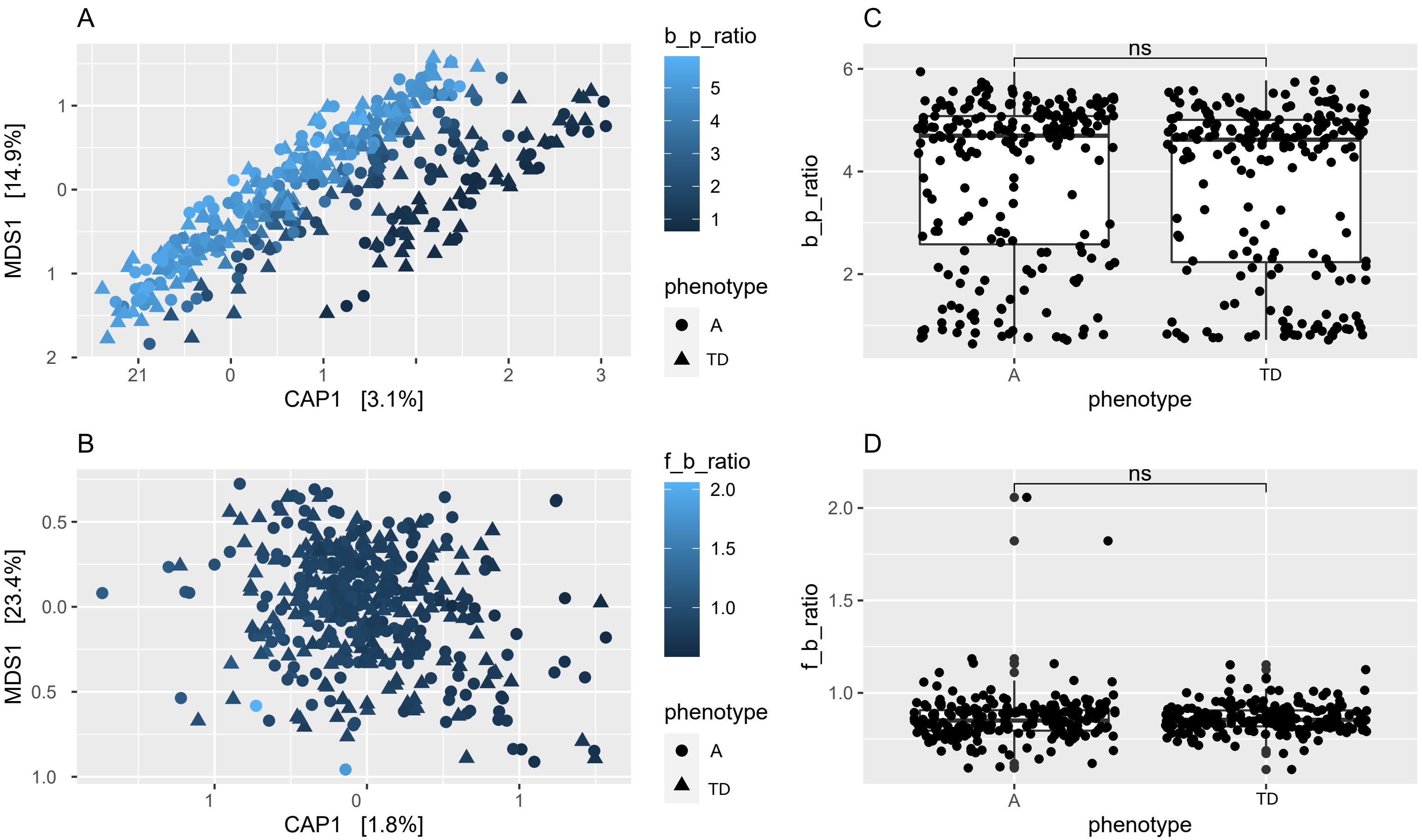

Supplementary File 12:
A) PCoA plot constrained by the ratio of Bacteroides to Prevotella
B) PCoA plot constrained by the ratio of Firmicutes to Bacteroidetes
C) Bacteroides to Prevotella ratio is not distinctly different between ASD and TD (rank sum test)
D) Firmicutes to Bacteroidetes ratio is not different between ASD and TD (rank sum test)

Supplementary File 13: Extended Materials/Subjects and Methods:

Autism Spectrum Disorder Diagnosis Verification

The Mobile Autism Risk Assessment (MARA), a brief level-2 screening measure for ASD, was collected from ASD participants [28]. The MARA is a behavioural questionnaire designed to screen children who are at high risk for ASD. Parents filled out the questionnaire about their children's behavior electronically and their answers were used in a classifier that outputs an ASD severity score [28]**.** Additionally, parents were asked to submit a short video of their child with and child without ASD enrolled in the study via encrypted file share. These videos were 2-6 minutes in length and were of the child during an independent or paired play session. These videos were then rated by minimally trained raters approved by the Stanford Institutional Review Board to score the presence and severity of a set of 30 behavioral features. Behavioral feature data collection methods are further described by Tariq et al [60].

The median of the scores across multiple raters were fed to previously published Machine Learning classifiers to predict ASD risk scores. [27,29]. By combining these risk scores with the parent-report screening tool (MARA), as well as parent-reported physician diagnosis, we confirmed diagnosis using majority rules consensus. We excluded (3) children and their TD siblings for whom the consensus did not agree with original parent diagnosis. We decided to include two children whose classifications did not reach majority consensus after realizing that the selected video-based classifiers did not include sensory dysregulation feature items. When viewing the sensory dysregulation item scores for these children, they scored in the ASD range, agreeing with their parent-reported diagnosis and their MARA scores.

Stool Collection and Storage

Stool samples were collected by the parents using a preservative buffer (Norgen Biotek, ON, Canada). The sampling consisted of 8 collection tubes. At the initial timepoint, we collected two samples per child: one sample was preserved at room temperature in a preservative buffer, and the second one was collected in a tube without a preservative buffer but immediately frozen at home at -20. This frozen sample was shipped back overnight with two ice packs provided to the participants. Other samples were shipped back every two weeks at room temperature in the preservative buffer. All shipping material was provided and pre-paid to the participants. Toilet collection containers were provided in the kit for collecting stool (sitting over the rear portion of the toilet). Participants were in contact with our clinical coordinator (by email or by phone). Once received, stool samples were stored at -80C until processing.

Sequence Processing, Filtering, and Taxonomic Annotation

Raw sequence reads were processed with DADA2 applying default settings for filtering, learning errors, dereplication, ASV inference, and chimera removal [62]. Truncation quality (truncQ) was set to 2. Ten nucleotides were then trimmed from each terminus of each read, both forward and reverse. An average of 156, 246 reads per sample library remained after processing the raw reads. For strain level ASV assignment, ASVs were mapped to an in-house strain database (StrainSelect,

https://www.secondgenome.com/platform/data-analysis-tools/strainselect, version 2019 (SS19)) using USEARCH (usearch_global) in the same manner as a recent study by Shah [63]. StrainSelect is a repository of strain identifiers obtained from gene sequencing, genome sequencing, draft genomes, and metagenomic assemblies, and assigns taxonomy using taxonomic annotations adapted from the Genome Taxonomy Database (GTDB). All sequences matching a unique strain at an identity ≥ 99% were assigned a strain-level annotation. To ensure specificity of these strain matches, a non-zero difference between the identity of the best match and the second best match was required *e.g.,* 99.75 vs. 99.5). Reads of the ASVs matched to the same strain were summed to represent the reads of the strain. If a unique strain match was not achieved, then species level and higher taxonomic placement was estimated with sintax (-cutoff 0.80) [64].

Normalization and Taxa Filtration

Before filtration, we attained a median read depth of 1.3 * 10^5 reads and a mean depth of 1.6*10^5 reads. Taxa not present in at least 3% of the samples were removed. Taxa abundances were normalized using DESeq2 or Cumulative Sum Scaling (CSS) depending on the contrast analysis performed. DESeq2 uses a negative binomial model and normalization by performing variance stabilization on taxa counts, then fitting a generalized linear model with log links to the normalized counts [65]. CSS normalizes counts by removing biases from taxa that are preferentially amplified in a sample-specific manner [66]. Due to how DESeq2 normalized minimized intra-group variance within families more so than CSS (ee Supplementary Information File 16), DESeq2 was used as the primary normalization in our gut-microbial community analysis.

In addition, taxa that significantly vary over time within the same individual were removed to increase the chance of identifying taxa directly related to core phenotype characteristics, rather than changes due to diet or season. A Friedman test was used to model ASV abundance as dependent on timepoint for each individual, and ASVs that were significantly related to timepoint ($p < .1$) were removed. 64 ASVs were removed from DESeq normalized data, 78 ASVs were removed from CSS normalized data, and 72 ASVs were removed from unnormalized data.

Analysis of Differentially Abundant Taxa between Phenotypes

ASV counts between the two phenotypes were compared using three common methods of differential analysis for amplicon data (DESeq2, MetagenomeSeq, and ANCOM2.1) [65, 66, 67]. Multiple analysis methods were employed to assess consistency of results and minimize spurious associations. In each of the above tests, we included the timepoint in the design matrix to account for differences between timepoints when estimating the effect of phenotype on any given taxa abundance.

Metadata Comparisons Between Cohorts

For each categorical variable from the metadata, a chi-square test was performed between the two cohorts. Wilcox-ranked sum tests were performed on non-longitudinal numerical metadata (age, general dietary habits, etc.) Two-way mixed repeated measures anovas were used on numerical metadata that were collected longitudinally.

Identifying Driving Factors in Gut-Microbial Community Structure

A PERMANOVA test consisting of the adonis and betadisper functions from the vegan package were used to assess whether or not metadata variables collected in this study via questionnaires had a significant association with the gut-microbial community structure of our samples. Permutations were performed with the strata option, in order to constraint permutation according to our repeated measure study design. This test was performed using Bray-Curtis distances and DESeq2 normalized counts. Variables with a significant adonis p-value (<0.05) and insignificant betadisper p-values were identified to be significant driving factors.

Identifying Confounding Factors impacting Microbial Structure

In order to address the potentially confounding variables, variables identified as driving factors in the PERMANOVA test and had significance in either a chi-square, wilcoxon-ranked sum test, or mixed two-way repeated measure ANOVA were also tested to find taxa that significantly differed based on these variables. MetagenomeSeq was used to identify specific ASVs associated with these confounding variables (Table 1). In addition, associations between beta-diversity and specific behavioral characteristics (MARA) were made only within the ASD cohort since TD individuals did not complete this questionnaire.

Summarizing Overall Effect Sizes of Lifestyle Variables vs. Microbial Features

In order to assess the overall scale of association between lifestyle, microbial factors, and ASD phenotype, we employed logistic regression using different feature sets. The features sets are composed as follows: 1) Basic (Age + Sex), 2) Basic + lifestyle/dietary variables, 3) Basic + microbiome features, 4) Basic + lifestyle/diet variables + microbiome features. Microbiome features were calculated as coordinates along a principal coordinate ordination using Bray Curtis distance. Regression models were built per timepoint (total of 3 timepoints) and 4-fold cross validation accuracy was reported over 4 random seeds. Changing the random seed resulted in different train/validation splits, and therefore contributed to the stability of the overall average estimate. Additionally, null models were created by drawing dummy variables from a uniform random distribution with minimum and maximum values corresponding to the true range of the variable. These dummy variables replaced the true variables in the models labeled "null" to represent the effect of non-informative patterns that may be captured due to random chance. A t-test was used to test the significance of differences in performance between models with different feature sets.

To determine whether statistically significant features from within the models were correlated with other variables not represented in the model, we performed a Pearson correlation between variables significantly associated with ASD and all other numerically quantified lifestyle variables. Association with ASD is reported as the Z-score within a linear model.

We then determined where the 11 biomarkers reported in Table1 fell along the axes of microbial variation (principal components) that were most significantly associated with ASD phenotypes. We used a modified Gene Set Enrichment Analysis (cite) where ASVs were ranked according to their

coordinates along a principal component, and two sets were created from biomarkers associated with ASD and TD phenotypes respectively.

Taxa Correlations with Anxiety Changes

Anxiety in the last 2 weeks was reported by caretakers on a scale of 1 to 3 where 1 meant low and 3 high anxiety. This metric was used to measure changes in anxiety within the same individual across time. We calculated the change in anxiety from timepoints 1 to 2, timepoints 2 to 3, and timepoints 1 to 3 for each individual. Samples without associated anxiety scores were removed. Respective changes in ASV abundance were calculated as log2 fold changes. We then correlated the log2 fold changes with changes in anxiety using a spearman correlation, and applied a multiple hypothesis correction. Positive values indicated an increase in anxiety or ASV abundance across timepoints for the same individual.

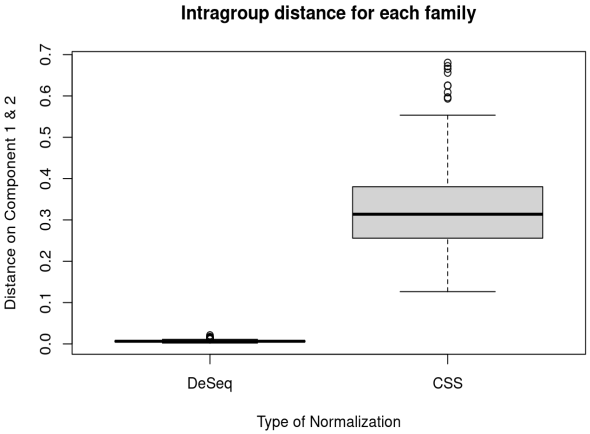

Supplementary File 14: Bray Curtis distance between samples from the same family. DESeq2minimizes the distance between samples expected to be close due to lifestyle, dietary, and physical proximity.
